# Anorectal emergencies: WSES-AAST guidelines

**DOI:** 10.1186/s13017-021-00384-x

**Published:** 2021-09-16

**Authors:** Antonio Tarasconi, Gennaro Perrone, Justin Davies, Raul Coimbra, Ernest Moore, Francesco Azzaroli, Hariscine Abongwa, Belinda De Simone, Gaetano Gallo, Giorgio Rossi, Fikri Abu-Zidan, Vanni Agnoletti, Gianluigi de’Angelis, Nicola de’Angelis, Luca Ansaloni, Gian Luca Baiocchi, Paolo Carcoforo, Marco Ceresoli, Alain Chichom-Mefire, Salomone Di Saverio, Federica Gaiani, Mario Giuffrida, Andreas Hecker, Kenji Inaba, Michael Kelly, Andrew Kirkpatrick, Yoram Kluger, Ari Leppäniemi, Andrey Litvin, Carlos Ordoñez, Vittoria Pattonieri, Andrew Peitzman, Manos Pikoulis, Boris Sakakushev, Massimo Sartelli, Vishal Shelat, Edward Tan, Mario Testini, George Velmahos, Imtiaz Wani, Dieter Weber, Walter Biffl, Federico Coccolini, Fausto Catena

**Affiliations:** 1 Emergency Surgery Department, Parma University Hospital, Parma, Italy; 2 Cambridge Colorectal Unit, Addenbrooke’s Hospital, Cambridge University Hospitals NHS Foundation Trust, Cambridge, UK; 3 Riverside University Health System Medical Center, Loma Linda University School of Medicine, Riverside, CA USA; 4 Ernest E. Moore Shock Trauma Center at Denver Health, Denver, CO USA; 5 Gastroenterology Unit, IRCCS Azienda Ospedaliero-Universitaria di Bologna, Department of Medical and Surgical Sciences (DIMEC), University of Bologna, Bologna, Italy; 6 Department of Metabolic, Digestive and Emergency Surgery, Centre Hospitalier Intercommunal de Poissy et Saint Germain en Laye, Poissy, France; 7 Department of Medical and Surgical Sciences, University of Catanzaro, Catanzaro, Italy; 8 Department of Surgery, College of Medicine and Health Sciences, UAE University, Al-Ain, United Arab Emirates; 9 Anesthesia and Intensive Care Unit, AUSL Romagna, M.Bufalini Hospital, Cesena, Italy; 10 Department of Medicine and Surgery, University of Parma, Parma, Italy; 11 Gastroenterology and Endoscopy Unit, Hospital of Parma, Parma, Italy; 12 Minimally Invasive and Robotic Digestive Surgery Unit, Regional General Hospital F. Miulli, Bari, Ital - Université Paris Est, UPEC, Creteil, France; 13 Department of Emergency and general Surgery, Pavia University Hospital, Pavia, Italy; 14 Department of Clinical and Experimental Sciences, University of Brescia, Brescia, Italy; 15 Department of Morphology, Surgery and Experimental Medicine, University of Ferrara, Ferrara, Italy; 16 General Surgery, Monza University Hospital, Monza, Italy; 17 Faculty of Health Sciences, Department of Surgery, University of Buea, Buea, Cameroon; 18 General surgery 1st unit, Department of General Surgery, University of Insubria, Varese, Italy; 19 Department of Medicine and Surgery, General Surgery Unit, University Hospital of Parma, Parma, Italy; 20 Department of General & Thoracic Surgery, University Hospital of Giessen, Giessen, Germany; 21 Division of Acute Care Surgery, University of Southern California, Los Angeles, CA USA; 22 Department of General Surgery, Albury Hospital, Albury, Australia; 23 General, Acute Care, Abdominal Wall Reconstruction, and Trauma Surgery, Foothills Medical Centre, Calgary, Alberta Canada; 24 Division of General Surgery, Rambam Health Care Campus, Haifa, Israel; 25 Helsinki University Hospital, Helsinki, Finland; 26 Department of Surgical Disciplines, Regional Clinical Hospital, Immanuel Kant Baltic Federal University, Kaliningrad, Russia; 27 Department of Surgery, Fundacion Valle del Lili - Universidad del Valle, Cali, Colombia; 28 University of Pittsburgh School of Medicine, UPMC-Presbyterian, Pittsburgh, PA USA; 29 3rd Department of Surgery, National & Kapodistrian University of Athens, Athens, Greece; 30 General Surgery Department, University Hospital St George, Plovdiv, Bulgaria; 31 Department of Surgery, Macerata Hospital, Macerata, Italy; 32 Department of Surgery, Tan Tock Seng Hospital, Singapore, Singapore; 33 Department of Surgery, Department of Emergency Medicine, Radboud University Medical Center, Nijmegen, the Netherlands; 34 Academic Unit of General Surgery “V. Bonomo” Department of Biomedical Sciences and Human Oncology, University of Bari, Bari, Italy; 35 Division of Trauma, Emergency Surgery, and Surgical Critical Care, Massachusetts General Hospital, Boston, MA USA; 36 Government Gousia Hospital, Srinagar, Kashmir India; 37 Department of General Surgery, Royal Perth Hospital, University of Western Australia, Perth, Australia; 38 Department of Trauma and Acute Care Surgery, Scripps Memorial Hospital La Jolla, La Jolla, San Diego, CA USA; 39 General, Emergency and Trauma Surgery Department, Pisa University Hospital, Pisa, Italy; 40 General, Emergency and Trauma Surgery Dept., Bufalini Hospital, Cesena, Italy

**Keywords:** Diagnosis, Non-operative management, Surgery, Antibiotics, Hemorrhoids, Fournier’s gangrene, Anorectal bleeding, Anorectal sepsis, Anorectal foreign bodies, Anorectal Varices, Technique, Timing, Angiography, Embolization, Guidelines

## Abstract

Anorectal emergencies comprise a wide variety of diseases that share common symptoms, i.e., anorectal pain or bleeding and might require immediate management. While most of the underlying conditions do not need inpatient management, some of them could be life-threatening and need prompt recognition and treatment. It is well known that an incorrect diagnosis is frequent for anorectal diseases and that a delayed diagnosis is related to an impaired outcome. This paper aims to improve the knowledge and the awareness on this specific topic and to provide a useful tool for every physician dealing with anorectal emergencies.

The present guidelines have been developed according to the GRADE methodology. To create these guidelines, a panel of experts was designed and charged by the boards of the World Society of Emergency Surgery (WSES) and American Association for the Surgery of Trauma (AAST) to perform a systematic review of the available literature and to provide evidence-based statements with immediate practical application. All the statements were presented and discussed during the WSES-AAST-WJES Consensus Conference on Anorectal Emergencies, and for each statement, a consensus among the WSES-AAST panel of experts was reached. We structured our work into seven main topics to cover the entire management of patients with anorectal emergencies and to provide an up-to-date, easy-to-use tool that can help physicians and surgeons during the decision-making process.

## Introduction

The definition of anorectal emergencies comprises a wide variety of diseases that share presenting symptoms, i.e., anorectal pain or bleeding, which may require immediate management. While most of the underlying conditions do not need inpatient management, some can be life-threatening, requiring prompt recognition and treatment. Making the correct initial diagnosis is probably the main issue in these cases, because the acute pain could preclude a complete and accurate clinical examination and the embarrassment related to the affected anatomical region delays the presentation of the patient in many cases. It is demonstrated that an incorrect recognition is frequent for anorectal diseases and that a delayed diagnosis is related to an adverse outcome [1]. Furthermore, anorectal disease remains a relevant healthcare problem that can consume a considerable amount of financial resource, both as direct costs related to medical treatments and as indirect expenses, such as loss of work days and impaired quality of life. In the light of the above , it becomes clear how important it is to spread knowledge in this field, improving diagnosis, treatment, and outcomes of such a common condition. Every physician working in an acute care setting will face this set of pathologies. This paper aims to improve the knowledge and the awareness on this specific topic and to provide a useful tool for every physician dealing with anorectal emergencies.

### Notes on the use of the guidelines: aims, targets, and limitations

These guidelines aim to present state of the art on diagnosis and therapeutic options for optimal management of anorectal emergencies. The intent is to improve the knowledge and the awareness of physicians around the world on this specific topic, providing an up-to-date tool that can help during the decision-making process. For this reason, the guidelines are evidence-based and the grade of recommendation is provided to summarize the evidence available in the literature. The population considered in these guidelines consists of adult patients with suspected anorectal emergencies. The practice guidelines promulgated in this work do not represent a standard of practice. They are suggested plans of care, based on the best available evidence and consensus of experts, but they do not exclude other approaches within the standard of practice. For example, they should not be used to compel adherence to a given method of medical management, which should be determined after taking into account the working conditions of each single medical institution (staff levels, experience, equipment, etc.) and the characteristics of the individual patient. However, the responsibility for the results of treatment rests with those who are directly engaged therein and not with the consensus group.

## Methods

To create these guidelines, a panel of experts was designed and charged by the boards of the World Society of Emergency Surgery (WSES) and of the American Association for the Surgery of Trauma (AAST), to develop questions on seven main topics that thoroughly cover the field of this pathology (retained anorectal bodies, perineal necrotizing fasciitis, complicated hemorrhoids, acute anal fissure, anorectal abscess, bleeding anorectal varices, complicated anorectal prolapse). Leading specialists in the field then were asked to perform a thorough search on each of these topics in different databanks (MEDLINE, SCOPUS, EMBASE) for relevant papers between 1985 and November 2019 and a systematic review of the available literature. They were asked to focus their search to provide evidence-based answers to every question with immediate practical application and to summarize them in statements. Furthermore, the references were kept up to date and the last literature search was performed in January 2021. All the statements were presented and discussed during the WSES - AAST - WJES Consensus Conference on Anorectal Emergencies held in Parma, Italy, on December 12, 2019, and for each statement, a consensus among the WSES - AAST panelists was reached. An international expert panel discussed the different issues in subsequent rounds. At each round, the manuscript was revised and improved. The final version about which agreement was reached resulted in the present manuscript. All the members contributed to the development of the manuscript.

The questions were created according to the PICO criteria, and the following guidelines adopt the GRADE methodology [2, 3].

### Topics and questions

For clarity, we report the seven topics together with the relative questions. The statements are summarized in Table 1.

**Table 1:** Statements

**Anorectal abscess**	
1.A - In patients with a suspected anorectal abscess, what is the role of clinical examination and biochemical investigations?	
	- In patients with suspected anorectal abscess, we suggest to collect a focused medical history and to perform a complete physical examination, including a digital rectal examination *(weak recommendation based on low-quality evidence, 2C).* - In patients with suspected anorectal abscess, we suggest to check serum glucose, hemoglobin a1c, and urine ketones in order to identify an undetected diabetes mellitus *(strong recommendation based on low-quality evidence, 1C).* - In patients with suspected anorectal abscess and signs of systemic infection or sepsis, we suggest to request complete blood count, serum creatinine, and inflammatory markers (e.g., C-reactive protein, procalcitonin, and lactates), to assess the status of the patient *(weak recommendation based on low-quality evidence, 2C).*
1.B - In patients with a suspected anorectal abscess, what are the appropriate imaging investigations?	
	- In patients with suspected anorectal abscess, we suggest the use of imaging investigations in case of atypical presentation and in case of suspicion of occult supralevator abscesses, complex anal fistula, or perianal Crohn’s disease. Suggested techniques are MRI, CT scan, or endosonography according to the specific clinical scenario and the available skills and resources *(weak recommendation based on low-quality evidence, 2C).*
1.C - In patients with an anorectal abscess, what are the indications for surgical treatment and what is the appropriate timing for surgery?	
	- In patients with anorectal abscess, we recommend a surgical approach with incision and drainage *(strong recommendation based on low-quality evidence, 1C).* - In patients with anorectal abscess, we suggest to base the timing of surgery on the presence and severity of sepsis* (weak recommendation based on low-quality evidence, 2C)*. - In fit, immunocompetent patients with a small perianal abscess and without systemic signs of sepsis, we suggest considering an outpatient management* (weak recommendation based on low-quality evidence, 2C)*.
1.D - In patients with an anorectal abscess, what is the role of wound packing after surgical drainage?	
	- No recommendation can be made regarding the use of packing after drainage of an anorectal abscess, based on the available literature.
1.E - In patients with an anorectal abscess and concomitant fistula, what are the indications for fistula treatment in the acute setting?	
	- In patients with anorectal abscess and an obvious fistula, we suggest to perform a fistulotomy at the time of abscess drainage only in cases of low fistula not involving sphincter muscle (i.e., subcutaneous fistula) *(weak recommendation based on low-quality evidence, 2C).* - In patients with anorectal abscess and an obvious fistula involving any sphincter muscle, we suggest to place a loose draining seton *(weak recommendation based on low-quality evidence, 2C).* - In patients with anorectal abscess and no obvious fistula, we suggest against probing to search for a possible fistula, to avoid iatrogenic complications *(weak recommendation based on low-quality evidence, 2C).*
1.F - In patients with an anorectal abscess, is there a role for antibiotic therapy and what is the appropriate antibiotic regimen?	
	- In patients with drained anorectal abscess, we suggest antibiotic administration in the presence of sepsis and/or surrounding soft tissue infection or in case of disturbances of the immune response *(weak recommendation based on low-quality evidence, 2C).* - In patients with anorectal abscess, we suggest sampling of drained pus in high-risk patients and/or in the presence of risk factors for multidrug-resistant organism infection *(weak recommendation based on very low-quality evidence, 2D).*
**Perineal necrotizing fasciitis (Fournier’s gangrene)**	
2.A - In patients with suspected Fournier’s gangrene, what is the role of clinical examination and biochemical investigations?	
	- In patients with suspected Fournier’s gangrene, we suggest to collect a focused medical history and a complete physical examination, including a digital rectal examination* (weak recommendation based on low-quality evidence, 2C).* - In patients with suspected Fournier’s gangrene and signs of systemic infection or sepsis, we suggest to request complete blood count and the dosage of serum creatinine and electrolytes, inflammatory markers (e.g., C-reactive protein, procalcitonin), and blood gas analysis, to assess the status of the patient *(weak recommendation based on low-quality evidence, 2C)*. We also recommend to check serum glucose, hemoglobin a1c and urine ketones in order to investigate an undetected diabetes mellitus *(strong recommendation based on low-quality evidence, 1C)*. - In patients with suspected Fournier’s gangrene, we suggest to use Laboratory Risk Indicator for Necrotising Fasciitis (LRINEC) score for an early diagnosis and Fournier’s Gangrene Severity Index (FGSI) for prognosis and risk stratification *(weak recommendation based on moderate quality evidence, 2B).*
2.B - In patients with suspected Fournier’s gangrene, which are the appropriate imaging investigations?	
	- In stable patients with suspected Fournier’s gangrene, we suggest to consider performing a CT scan *(weak recommendation based on low-quality evidence, 2C).* - In patients with Fournier’s gangrene, we recommend that imaging should not delay surgical intervention *(strong recommendation based on moderate quality evidence, 1B).* - In patients with Fournier’s gangrene and hemodynamic instability persisting after proper resuscitation, we suggest against CT imaging *(weak recommendation based on low-quality evidence, 2C).*
2.C - In patients with Fournier’s gangrene, what are the indications for surgical treatment and what is the appropriate timing for surgery?	
	- In patients with Fournier’s gangrene, we recommend surgical intervention as soon as possible *(strong recommendation based on low-quality evidence, 1C)*. - In patients with Fournier’s gangrene we suggest planning repeat surgical revisions (exploration and debridement) according to patient conditions *(weak recommendation based on low-quality evidence, 2C)*. - In patients with Fournier’s gangrene, we suggest seriated surgical revisions until the patient is free of necrotic tissue *(weak recommendation based on low-quality evidence, 2C).*
2.D - In patients with Fournier’s gangrene, what is the appropriate surgical approach?	
	- In patients with Fournier’s gangrene, we suggest to remove all the necrotic tissue *(weak recommendation based on low-quality evidence, 2C)*. - In patients with Fournier’s gangrene, we suggest a multidisciplinary and tailored approach based upon the extent of perineal involvement, the degree of fecal contamination, and the possible presence of sphincter or urethral damage *(weak recommendation based on low-quality evidence, 2C).* - In patients with Fournier’s gangrene, we suggest to perform orchiectomy or other genital surgery only if strictly necessary and possibly based on a urologic consultation *(weak recommendation based on low-quality evidence, 2C).* - In patients with Fournier’s gangrene, we suggest planning the surgical management of early and delayed surgical sequelae with a multidisciplinary and skilled team *(weak recommendation based on low-quality evidence, 2C).*
2.E - In patients with Fournier’s gangrene, which is the appropriate antibiotic regimen?	
	- In patients with Fournier’s gangrene, we recommend starting an empiric antimicrobial therapy as soon as the diagnosis is suspected *(strong recommendation based on moderate quality evidence, 1B).* - In patients with Fournier’s gangrene, we recommend that empiric antimicrobial therapy should include cover for gram-positive, gram-negative, aerobic and anaerobic bacteria, and an anti-MRSA agent *(strong recommendation based on moderate quality evidence, 1B).* - In patients with Fournier’s gangrene, we recommend to obtain microbiological samples at the index operation *(strong recommendation based on moderate quality evidence, 1B).* - In patients with Fournier’s gangrene, we recommend to base antimicrobial de-escalation on clinical improvement, cultured pathogens, and results of rapid diagnostic tests where available *(strong recommendation based on moderate quality evidence, 1B).*
**Complicated hemorrhoid (thrombosed, strangulated, or bleeding)**	
3.A - In patients with suspected complicated hemorrhoids, what is the role of clinical examination and biochemical investigations?	
	- No recommendation can be made regarding the role of biochemical investigations in patients with suspected thrombosed or strangulated hemorrhoids, based on the available literature. - In patients with suspected bleeding hemorrhoids, we suggest to collect a focused medical history and to perform a complete physical examination, including a digital rectal examination, to rule out other causes of lower gastrointestinal bleeding *(weak recommendation based on low-quality evidence, 2C).* - In patients with suspected bleeding hemorrhoids, we suggest to check vital signs, to determine hemoglobin and hematocrit, and to assess coagulation to evaluate the severity of the bleeding *(weak recommendation based on low-quality evidence, 2C).* In case of severe bleeding, we suggest blood typing and cross-matching *(weak recommendation based on very low-quality evidence, 2D).*
3.B - In patients with suspected complicated hemorrhoids, which are the appropriate imaging investigations?	
	- In patients with suspected complicated hemorrhoids, we suggest to perform imaging investigation (CT scan, MRI, or endoanal ultrasound) only if there is suspicion of concomitant anorectal diseases (sepsis/abscess, inflammatory bowel disease, neoplasm) *(weak recommendation based on low-quality evidence, 2C).*
3.C - In patients with complicated hemorrhoids, what is the role of endoscopy?	
	- In patients with complicated hemorrhoids, we suggest to perform anoscopy as part of the physical examination, whenever feasible and well tolerated *(low recommendation based on low-quality evidence, 2C).* - In patients with complicated hemorrhoids, we suggest to perform colonoscopy in case of concern for inflammatory bowel disease or cancer arising from patient personal and family history, or from physical examination *(low recommendation based on low-quality evidence, 2C).*
3.D - In patients with complicated hemorrhoids, which is the appropriate pharmacological regimen?	
	- In patients with complicated hemorrhoids, we recommend non-operative management as first line therapy, with dietary and lifestyle changes (i.e., increased fiber and water intake together with adequate bathroom habits) *(strong recommendation based on moderate quality evidence, 1B).* - In patients with complicated hemorrhoids, we suggest to administer flavonoids to relieve symptoms *(weak recommendation based on moderate quality evidence, 2B).* - In patients with thrombosed or strangulated hemorrhoids, we suggest the use of topical muscle relaxant *(weak recommendation based on low-quality evidence, 2C).* - No recommendation can be made regarding the role of NSAIDs, topical steroids, other topical agents, or injection of local anesthetics for complicated hemorrhoids, based on the available literature.
3.E - In patients with complicated hemorrhoids, what is the role of office-based procedures?	
	- No recommendation can be made regarding the role of office-based procedures (i.e., rubber band ligation, sclerotherapy, infrared coagulation) in complicated hemorrhoids, based on the available literature.
3.F - In patients with complicated hemorrhoids, what are the indications for surgical treatment and what is the appropriate timing for surgery?	
	- In patients with thrombosed hemorrhoids, we suggest to base the decision between non-operative management and early surgical excision on local expertise and patient’s preference *(weak recommendation based on low-quality evidence, 2C).* - In patients with thrombosed hemorrhoids, we suggest against the use of incision and drainage of the thrombus *(weak recommendation based on low-quality evidence, 2C).* - No recommendation can be made regarding the role of surgery in patients with bleeding hemorrhoids, based on the available literature.
3.G - In patients with complicated hemorrhoids, what is the role of angiography?	
	- No recommendation can be made regarding the role of angiography in complicated hemorrhoids, based on the available literature.
**Bleeding anorectal varices**	
4.A - In patients with suspected bleeding anorectal varices, what is the role of clinical examination and biochemical investigations?	
	- In patients with suspected bleeding anorectal varices, we suggest to collect a focused medical history and to perform a complete physical examination, including a digital rectal examination, to rule out other causes of lower gastrointestinal bleeding *(weak recommendation based on low-quality evidence, 2C).* - In patients with suspected anorectal varices, we suggest we suggest to check vital signs, to determine hemoglobin and hematocrit, and to assess coagulation to evaluate the severity of the bleeding *(weak recommendation based on low-quality evidence, 2C)*. In case of severe bleeding, we suggest blood typing and cross-matching *(weak recommendation based on very low-quality evidence, 2D).*
4.B - In patients with suspected bleeding anorectal varices, which are the appropriate imaging investigations?	
	- In patients with bleeding anorectal varices, we suggest EUS +/- color Doppler evaluation as a second-line diagnostic tool, especially for deep rectal varices or when in doubt *(weak recommendation based on low-quality evidence, 2C).* - In patients with bleeding anorectal varices and failed detection of bleeding site at endoscopy and EUS, or whenever EUS is not available, we suggest to perform contrast enhanced CT-scan *(weak recommendation based on low-quality evidence, 2C).* - In pregnant patients with bleeding anorectal varices and failed US detection of bleeding site, we suggest to perform MRI angiography, if available and if allowed by the clinical scenario *(weak recommendation based on low-quality evidence, 2C).*
4.C - In patients with suspected bleeding anorectal varices, what is the role of endoscopy?	
	- In patients with suspected bleeding anorectal varices, we suggest the use of ano-proctoscopy or flexible sigmoidoscopy as the first-line diagnostic tool *(weak recommendation based on low-quality evidence, 2C).* - In patients with suspected bleeding anorectal varices and high-risk features or evidence of ongoing bleeding, we suggest to perform an urgent colonoscopy (plus upper endoscopy) within 24 hours of presentation *(weak recommendation based on low-quality evidence, 2C).* - In patients with suspected bleeding anorectal varices and risk factors for colorectal cancer or suspicion of a concomitant more proximal source of bleeding, we suggest to perform a full colonoscopy *(weak recommendation based on low-quality evidence, 2C).* - In patients with bleeding anorectal varices, we suggest to use local procedures, such as endoscopic variceal ligation, endoscopic band ligation, sclerotherapy, or EUS-guided glue injection, to arrest bleeding in first instance where feasible *(weak recommendation based on low-quality evidence, 2C).*
4.D - In patients with bleeding anorectal varices, what is the role of non operative management?	
	- In patients with bleeding anorectal varices, we suggest multidisciplinary management, early involving the hepatology specialist team and focusing on optimal control of comorbid conditions *(weak recommendation based on very low-quality evidence, 2D).* - In patients with anorectal varices and mild bleeding, we suggest intravenous fluid replacement, blood transfusion if necessary, correction of coagulopathy, and optimal medication for portal hypertension *(weak recommendation based on low-quality evidence, 2C).* - In patients with anorectal varices and severe bleeding, we recommend to maintain an Hb level of at least > 7 g/dl (4.5 mmol/l) during the resuscitation phase and a mean arterial pressure > 65 mmhg, but avoiding fluid overload *(strong recommendation based on moderate quality evidence, 1B).* - In patients with bleeding anorectal varices, we suggest the endorectal placement of a compression tube as a bridging maneuver, to help stabilization of the patient or to allow the transfer to a tertiary hospital *(weak recommendation based on very low-quality evidence, 2D)*.
4.E - In patients with bleeding anorectal varices, which is the appropriate pharmacological regimen (including antibiotics)?	
	- In patients with anorectal varices, we suggest the use of non-selective beta-adrenergic blockers for prevention/prophylaxis of first and/or recurrent variceal bleeding *(weak recommendation based on very low-quality evidence, 2D)*. In case of acute bleeding, we suggest to temporarily suspended beta blockers *(weak recommendation based on low-quality evidence, 2C).* - In patients with bleeding anorectal varices, we suggest to consider the use of vasoactive drugs, such as terlipressin or octreotide, to reduce splanchnic blood flow and portal pressure *(weak recommendation based on very low-quality evidence, 2D).* - In patients with bleeding anorectal varices, we recommend a short course of prophylactic antibiotic* (strong recommendation based on moderate quality evidence, 1B).*
4.F - In patients with bleeding anorectal varices, what is the role for angiography?	
	- In patients with bleeding anorectal varices and failure of medical treatment and local procedures, we suggest a “step up” approach with radiological and then surgical procedures* (weak recommendation based on low-quality evidence, 2C).* - In patients with bleeding anorectal varices, we suggest to use embolization via interventional radiological techniques for the short-term control of bleeding *(weak recommendation based on low-quality evidence, 2C).* - In patients with bleeding anorectal varices and severe portal hypertension, we suggest to use percutaneous TIPS, if not contraindicated, to decompress the portal venous system and to reduce the risk for rebleeding *(weak recommendation based on low-quality evidence, 2C).* - No recommendation can be made regarding the superiority of one embolization technique over the others in case of bleeding anorectal varices , based on the available literature.
4.G - In patients with bleeding anorectal varices, what are the indications for surgical treatment and what is the appropriate timing for surgery?	
	- In patients with bleeding anorectal varices and failure of medical treatment, local and radiological procedures, we suggest a “step up” approach with surgical procedures *(weak recommendation based on low-quality evidence, 2C).* - In patients with bleeding anorectal varices and failure of medical treatment, local and radiological procedures, we suggest against the use of “per anal” suture ligation *(weak recommendation based on very low-quality evidence, 2D).* - No recommendation can be made regarding the role of Doppler-guided hemorrhoidal artery ligation and stapled anopexy in patients with bleeding anorectal varices and failure of medical treatment,local and radiological procedures, based on the available literature.
**Complicated rectal prolapse (irreducible or strangulated)**	
5.A - In patients with a suspected complicated rectal prolapse, what is the role of clinical examination and biochemical investigations?	
	- In patients with suspected complicated rectal prolapse, we suggest to request complete blood count and the dosage of serum creatinine, and inflammatory markers (e.g., C-reactive protein, procalcitonin, and lactates) to assess the status of the patient *(weak recommendation based on low-quality evidence, 2C).*
5.B - In patients with a suspected complicated rectal prolapse, which are the appropriate imaging investigations?	
	- In hemodynamically stable patients with irreducible or strangulated rectal prolapse, we suggest to perform an urgent contrast enhanced abdomino-pelvic CT-scan, whenever available and without delaying appropriate treatment, to detect possible associated complications and to assess the presence of a colorectal cancer *(weak recommendation based on low-quality evidence, 2C).* - In hemodynamically unstable patients with irreducible or strangulated rectal prolapse, we suggest against delaying appropriate and timely management to perform imaging investigations *(weak recommendation based on low-quality evidence, 2C).*
5.C - In patients with complicated rectal prolapse, what is the role of non-operative management?	
	- In patients with incarcerated rectal prolapse without signs of ischemia or perforation, we suggest to attempt conservative measures and gentle manual reduction under mild sedation or anesthesia *(weak recommendation based on moderate quality evidence, 2B).* - In hemodynamically unstable patients with complicated rectal prolapse, we suggest against delaying surgical management to attempt a conservative management *(weak recommendation based on low-quality evidence, 2C).*
5.D - In patients with complicated rectal prolapse, what are the indications for surgical treatment and what is the appropriate timing for surgery?	
	- In patients with complicated rectal prolapse and signs of shock or gangrene/perforation of prolapsed bowel, we recommend immediate surgical treatment *(strong recommendation based on high-quality evidence, 1A).* - In patients with complicated rectal prolapse and bleeding, acute bowel obstruction or failure of non-operative management, we suggest urgent surgical treatment *(weak recommendation based on low-quality evidence, 2C).*
5.E - In patients with complicated rectal prolapse, what is the most appropriate surgical approach?	
	- In patients with complicated rectal prolapse and no signs of peritonitis or hemodynamic instability, we suggest to base the decision between abdominal and perineal procedures on the specific patient’s characteristics and on surgeon’s skills and expertise *(weak recommendation based on moderate quality evidence, 2B)*. - In hemodynamically stable patients with complicated rectal prolapse, in case of abdominal approach, we suggest to base the decision between open or laparoscopic surgery on patient’s characteristics and on surgeon’s skills and expertise *(weak recommendation based on very low-quality evidence, 2D).* - In patients with complicated rectal prolapse and signs of peritonitis, we suggest an abdominal approach *(weak recommendation based on low-quality evidence, 2C)*. - In patients with complicated rectal prolapse and hemodynamic instability, we recommend an abdominal open approach* (strong recommendation based on low-quality evidence, 1C)*. - In patients with complicated rectal prolapse undergoing resectional surgery, we suggest to base the decision between primary anastomosis, with or without diverting ostomy, and terminal colostomy on the patient’s clinical condition and on the individual risk of anastomotic leakage *(weak recommendation based on low-quality evidence, 2C).*
5.F - In patients with complicated rectal prolapse, which is the appropriate pharmacological regimen (antibiotics, pain-control, others)?	
	- In patients with strangulated rectal prolapse, we suggest to administer empiric antimicrobial therapy because of the risk of intestinal bacterial translocation; the appropriate regimen should be based on the clinical condition of the patients, the individual risk for MDRO, and the local resistance epidemiology *(weak recommendation based on low-quality evidence, 2C).*
**Retained anorectal foreign bodies**	
6.A - In patients with a suspected retained anorectal foreign body, what is the role of clinical examination and biochemical investigations?	
	- In patients with suspected retained anorectal foreign body, we suggest to collect a focused medical history and to perform a complete physical examination *(weak recommendation based on low-quality evidence, 2C).* - In patients with suspected retained anorectal foreign body, we suggest to perform digital rectal examination after the acquisition of an abdomen X-ray, whenever possible, to prevent accidental injury to the surgeon from sharp objects *(weak recommendation based on low-quality evidence, 2C).* - In patients with suspected retained anorectal foreign body and no signs of bowel perforation, we suggest against routinely requesting of laboratory tests *(weak recommendation based on very low-quality evidence, 2D).* - In patients with suspected retained anorectal foreign body and no signs of bowel perforation, we suggest to request the routine preoperative blood tests only in case manual extraction fails/is not feasible *(weak recommendation based on very low-quality evidence, 2D).* - In patients with suspected retained anorectal foreign body and coexisting suspected bowel perforation, we suggest to request complete blood count and the dosage of serum creatinine, and inflammatory markers (e.g., C-reactive protein, procalcitonin, and lactates) to assess the status of the patient prior to surgery* (weak recommendation based on low-quality evidence, 2C)*
6.B - In patients with a suspected retained anorectal foreign body, which are the appropriate imaging investigations?	
	- In patients with suspected retained anorectal foreign body, we recommend lateral and anteroposterior plain X-ray film of the chest, abdomen, and pelvis to identify the foreign body position and determine its shape, size, and location and the possible presence of pneumoperitoneum *(strong recommendation based on moderate quality evidence, 1B).* - In hemodynamically stable patients with suspected retained anorectal foreign body and a suspected perforation, we recommend a contrast enhanced CT scan of the abdomen *(strong recommendation based on moderate quality evidence, 1B).* - In patients with retained anorectal foreign body and hemodynamic instability, we suggest against delaying surgical treatment to perform imaging investigations *(weak recommendation based on low-quality evidence, 2C).*
6.C - In patients with a retained anorectal foreign body, what is the most appropriate interventional approach (manual extraction vs endoscopy vs surgery)?	
	- In patients with low-lying retained anorectal foreign body without sign of perforation, we suggest an attempt of bedside extraction as the first-line therapy *(weak recommendation based on low-quality evidence, 2C).* - No recommendation can be made regarding the superiority of one trans-anal extraction technique over the others in case of retained anorectal foreign body, based on the available literature. - In patients with retained anorectal foreign body and failure of bedside extraction, we suggest pudendal nerve block, spinal anesthesia, intravenous conscious sedation, or general anesthesia to improve chances of transanal retrieval *(weak recommendation based on low-quality evidence, 2C).* - In patients with retained high-lying anorectal foreign body (above rectosigmoid junction), we suggest an attempt of endoscopic extraction as the first-line therapy *(weak recommendation based on low-quality evidence, 2C).* - In patients with retained anorectal foreign body and suspect of drug concealment, we suggest against any maneuver that can disrupt the drug package, including endoscopic retrieval *(weak recommendation based on low-quality evidence, 2C).* - In patients with retained anorectal foreign body, we suggest to perform a proctoscopy or flexible sigmoidoscopy after foreign body removal, to evaluate bowel wall status *(weak recommendation based on low-quality evidence, 2C).* - In patients with retained anorectal foreign body and signs and hemodynamic instability or perforation, we recommend against transanal extraction *(strong recommendation based on low-quality evidence, 1C).*
6.D - In patients with a retained anorectal foreign body, what are the indications for surgical treatment and what is the appropriate timing for surgery?	
	- In patients with retained anorectal foreign body and no signs of perforation, we suggest a surgical approach in case of failure of transanal extraction* (weak recommendation based on low-quality evidence, 2C)*. - In patients with retained anorectal foreign body and no signs of perforation, we suggest a “step-up” surgical approach, starting with downward milking and proceeding to colotomy only when milking/transanal extraction fails *(weak recommendation based on low-quality evidence, 2C).* - In patients with retained anorectal foreign body and no signs of perforation, we suggest a laparoscopic approach if skills and instrumentation are available *(weak recommendation based on low-quality evidence, 2C).* - In patients with retained anorectal foreign body and bowel perforation with limited peritoneal contamination, we suggest primary suture only in case of small and recent perforation and if the colonic tissues appear healthy and well vascularized, and an approximation of perforation edges could be performed without tension *(weak recommendation based on low-quality evidence, 2C)* - In patients with retained anorectal foreign body and bowel perforation, clinically stable and without risk factors for anastomotic leakage, when primary suture is not feasible, we suggest resection with primary anastomosis with or without a diverting stoma *(weak recommendation based on low-quality evidence, 2C)* - In critically ill patients with retained anorectal foreign body and bowel perforation, or in selected patients with extensive peritoneal contamination and risk factors for anastomotic leakage, we suggest to perform a Hartmann’s procedure *(weak recommendation based on low quality evidence, 2C)* - In patients with retained anorectal foreign body and hemodynamic instability, we recommend an emergent laparotomy and a damage control surgery approach *(strong recommendation based on moderate quality evidence, 1B).*
6.E - In patients with a retained anorectal foreign body, is there a role for antibiotic therapy?	
	- In patients with retained anorectal foreign body, we suggest against the routinely use of antimicrobial therapy *(weak recommendation based on low-quality evidence, 2C).* - In patients with retained anorectal foreign body and signs of hemodynamic instability or perforation, we recommend broad spectrum antibiotic therapy according to the WSES guidelines on intra-abdominal infections *(strong recommendation based on moderate quality evidence, 1B).*
**Acute anal fissure**	
7.A - In patients with suspected acute anal fissure, what is the role of clinical examination and biochemical investigations?	
	- No recommendation can be made regarding the role of biochemical investigations in patients with typical acute anal fissure, based on the available literature - In patients with atypical acute anal fissure, we suggest to collect a focused medical history, perform a complete physical examination and laboratory tests based on the suspected associated illness, to rule out other causes *(weak recommendation based on low-quality evidence, 2C).*
7.B - In patients with suspected acute anal fissure, which are the appropriate imaging investigations?	
	- No recommendation can be made regarding the use of imaging investigations in patients with typical acute anal fissure, based on the available literature. - In patients with atypical acute anal fissure, we suggest to perform investigations (endoscopy, CT scan, MRI, or endoanal ultrasound) only in case of suspected concomitant inflammatory bowel disease,anal or colorectal cancer or occult perianal sepsis *(weak recommendation based on low-quality evidence, 2C).*
7.C - In patients with an acute anal fissure, what is the role of non-operative management?	
	- In patients with acute anal fissure, we recommend non-operative management as the first-line treatment *(Strong recommendation based on moderate quality evidence, 1B)* - In patients with acute anal fissure, we recommend dietary and lifestyle changes, with increased fiber and water intake *(strong recommendation based on moderate quality evidences, 1B)*. - In patients with acute anal fissure, we recommend against the use of manual dilatation *(strong recommendation based on moderate quality evidences, 1B)*. - No recommendation can be made regarding the use of controlled anal dilatation in patients with acute anal fissure, based on the available literature.
7.D - In patients with an acute anal fissure, what is the appropriate approach for pain control?	
	- In patients with acute anal fissure, we suggest the integration of topical anesthetics and common pain killers in case of inadequate pain control *(weak recommendation based on low-quality evidences, 2C).* - No recommendation can be made regarding the use of botulinum injections in patients with acute anal fissure, based on the available literature.
7.E - In patients with an acute anal fissure, is there a role for antibiotic therapy?	
	- In patients with acute anal fissure, we suggest the use of topical antibiotics in case of potential reduced therapeutic compliance or poor genital hygiene *(weak recommendation based on very low-quality evidences, 2D).*
7.F - In patients with an acute anal fissure, what are the indications for surgical treatment and what is the appropriate timing for surgery? If indicated, what is the most appropriate surgical approach?	
	- In patients with acute anal fissure, we suggest against surgical treatment *(weak recommendation based on moderate quality evidences, 2B).* - In patients with anal fissure, we suggest surgical treatment in the chronic phase, if non responsive after 8 weeks non-operative management *(strong recommendation based on moderate quality evidences, 1B).*

#### 1) Anorectal abscess

A. In patients with a suspected anorectal abscess, what is the role of clinical examination and biochemical investigations?
B. In patients with a suspected anorectal abscess, what are the appropriate imaging investigations?
C. In patients with an anorectal abscess, what are the indications for surgical treatment and what is the appropriate timing for surgery?
D. In patients with an anorectal abscess, what is the role of wound packing after surgical drainage?
E. In patients with an anorectal abscess and concomitant fistula, what are the indications for fistula treatment in the acute setting?
F. In patients with an anorectal abscess, is there a role for antibiotic therapy and what is the appropriate antibiotic regimen?

#### 2) Perineal necrotizing fasciitis (Fournier’s gangrene)

A. In patients with suspected Fournier’s gangrene, what is the role of clinical examination and biochemical investigations?
B. In patients with suspected Fournier’s gangrene, which are the appropriate imaging investigations?
C. In patients with Fournier’s gangrene, what are the indications for surgical treatment and what is the appropriate timing for surgery?
D. In patients with Fournier’s gangrene, what is the most appropriate surgical approach?
E. In patients with Fournier’s gangrene, which is the appropriate antibiotic regimen?

#### 3) Complicated hemorrhoids (thrombosed, strangulated, or bleeding)

A. In patients with suspected complicated hemorrhoids, what is the role of clinical examination and biochemical investigations?
B. In patients with suspected complicated hemorrhoids, which are the appropriate imaging investigations?
C. In patients with complicated hemorrhoids, what is the role of endoscopy?
D. In patients with complicated hemorrhoids, which is the appropriate pharmacological regimen?
E. In patients with complicated hemorrhoids, what is the role of office-based procedures?
F. In patients with complicated hemorrhoids, what are the indications for surgical treatment and what is the appropriate timing for surgery?
G. In patients with complicated hemorrhoids, what is the role of angiography?

#### 4) Bleeding anorectal varices

A. In patients with suspected bleeding anorectal varices, what is the role of clinical examination and biochemical investigations?
B. In patients with suspected bleeding anorectal varices, which are the appropriate imaging investigations?
C. In patients with suspected bleeding anorectal varices, what is the role of endoscopy? D In patients with bleeding anorectal varices, what is the role of non-operative management?
D. E. In patients with bleeding anorectal varices, which is the appropriate pharmacological regimen (including antibiotics)?
E. F. In patients with bleeding anorectal varices, what is the role for angiography?
F. G. In patients with bleeding anorectal varices, what are the indications for surgical treatment and what is the appropriate timing for surgery?

#### 5) Complicated rectal prolapse (irreducible or strangulated)

A. In patients with suspected complicated rectal prolapse, what is the role of clinical examination and biochemical investigations?
B. In patients with suspected complicated rectal prolapse, which are the appropriate imaging investigations?
C. In patients with complicated rectal prolapse, what is the role of non-operative management?
D. In patients with complicated rectal prolapse, what are the indications for surgical treatment and what is the appropriate timing for surgery?
E. In patients with complicated rectal prolapse, what is the most appropriate surgical approach?
F. In patients with complicated rectal prolapse, which is the appropriate pharmacological regimen (antibiotics, pain-control, others)?

#### 6) Retained anorectal foreign bodies

A. In patients with a suspected retained anorectal foreign body, what is the role of clinical examination and biochemical investigations?
B. In patients with a suspected retained anorectal foreign body, which are the appropriate imaging investigations?
C. In patients with a retained anorectal foreign body, what is the most appropriate interventional approach (manual extraction vs endoscopy vs surgery)?
D. In patients with a retained anorectal foreign body, what are the indications for surgical treatment and what is the appropriate timing for surgery?
E. In patients with a retained anorectal foreign body, is there a role for antibiotic therapy?

#### 7) Acute anal fissure

A. In patients with a suspected acute anal fissure, what is the role of clinical examination and biochemical investigations?
B. In patients with a suspected acute anal fissure, which are the appropriate imaging investigations?
C. In patients with an acute anal fissure, what is the role of non-operative management?
D. In patients with an acute anal fissure, what is the appropriate approach for pain control?
E. In patients with an acute anal fissure, is there a role for antibiotic therapy?
F. In patients with an acute anal fissure, what are the indications for surgical treatment and what is the appropriate timing for surgery? If indicated, what is the most appropriate surgical approach?

## Guidelines

1. Anorectal abscess
  **1.A - In patients with a suspected anorectal abscess, what is the role of clinical examination and biochemical investigations?**

*In patients with suspected anorectal abscess, we suggest to collect a focused medical history and to perform a complete physical examination, including a digital rectal examination (weak recommendation based on low-quality evidence, 2C).*

*In patients with suspected anorectal abscess, we suggest to check serum glucose, hemoglobin a1c, and urine ketones in order to identify an undetected diabetes mellitus (strong recommendation based on low-quality evidence, 1C).*

*In patients with suspected anorectal abscess and signs of systemic infection or sepsis, we suggest to request a complete blood count, serum creatinine, and inflammatory markers (e.g., C-reactive protein, procalcitonin, and lactates), to assess the status of the patient (weak recommendation based on low-quality evidence, 2C).*

An anorectal abscess is characterized by an infection in the soft tissue around the anus. However, depending on the different anatomical locations of the infectious process, the manifestations, and the treatment of anorectal abscesses vary widely. It is important to remember the anatomical classification of intersphincteric, perianal, ischiorectal, or supralevator abscesses [4]. Anorectal abscesses are associated with anal fistulas in approximately a third of patients [5], and the classification of fistulae depends on their anatomical location as well. The clinical presentation is typically characterized by perianal pain, swelling, and fever, but deeper abscesses may also present with pain referred to the perineum, low back, and buttocks or with symptoms that mimic an intra-abdominal condition; discharge of pus could also be present and occasionally patients with anorectal abscesses will present with urinary retention [6]. It is a common disease and constitutes a common presentation to emergency general surgeons; the most common age for presentation is 20–40 years, with men more commonly affected than women [7, 8]. The majority are idiopathic in nature, with the cryptoglandular hypothesis being most commonly accepted, but a proportion may present as part of another disease process, most commonly Crohn’s disease. The differential diagnosis of anorectal abscesses is wide and comprises other significant diseases such as anal cancer and precancerous conditions, Crohn’s disease, and tuberculosis. In fact, an anorectal abscess will develop in approximately one third of patients with Crohn’s disease [9]. Furthermore, it is important to make an early distinction from Fournier’s gangrene/necrotizing fasciitis. The most common anatomical locations are perianal and ischio-rectal, with intersphincteric and supralevator being less common. The management of patients with known Crohn’s disease goes beyond the aims of these guidelines, but it is mandatory to exclude the presence of undiagnosed underlying Crohn’s disease in every patient presenting with an anorectal abscess, especially if recurrent [10]. For this reason, a focused and detailed medical history is of utmost importance and complete physical examination should include a careful inspection of the perineum checking for surgical scars, anorectal deformities, other signs of perianal Crohn’s disease, the presence of secondary cellulitis, or the presence of external opening of an anal fistula. A complete anorectal examination, including digital rectal examination, is usually feasible, but in some cases, sedation or anesthesia may be needed due to the intense pain. The patient’s history and physical examination are usually enough to diagnose small and superficial abscesses [11] , while deeper abscesses can be challenging to diagnose; in these cases, rectal examination usually reveals a tender, indurated area above the anorectal ring.

Laboratory and radiological studies are not usually needed for the diagnosis of an anorectal abscess but can be useful in specific situations. The decision to perform laboratory tests to assess the severity of illness should be guided by the clinical findings. Routine laboratory tests can provide an insight on the general health status of the patient and can help physicians understand the severity of the situation, especially in case of potential candidates for emergency surgery or with signs of hemodynamic instability.

It is important to remember that symptoms are frequently absent or diminished in older and debilitated patients, in patients with diabetes or other forms of immunosuppression, and in some cases of associated necrotizing soft-tissue infection [11]: for this reason, the treatment of these subsets of patients requires a great degree of suspicion and an aggressive approach.

- **1.B - In patients with a suspected anorectal abscess, what are the appropriate imaging investigations?**

*In patients with suspected anorectal abscess, we suggest the use of imaging investigations in case of atypical presentation and in case of suspicion of occult supralevator abscesses, complex anal fistula, or perianal Crohn’s disease. Suggested techniques are MRI, CT scan, or endosonography according to the specific clinical scenario and the available skills and resources (weak recommendation based on low-quality evidence, 2C).*

Radiological studies are not usually needed for the diagnosis of an anorectal abscess but can be useful in some special situations. The use of imaging techniques could be helpful in all those cases with an atypical presentation (e.g., lower back pain, severe anal pain in the absence of a fissure, urinary retention), when the physical examination suggests a supralevator or intersphincteric abscess or when there is suspicion of perianal Crohn’s disease. Fistulography used to be the method of choice for the radiological assessment of fistula-in-ano, but its accuracy rate could be as low as 16% [12] and nowadays has been replaced by more accurate imaging techniques.

MRI has high detection rates for anorectal abscesses [13], while there is debate around the sensitivity and specificity of anal endosonography (EUS). Some studies suggest that EUS is more accurate than MRI in detecting abscesses and in the evaluation of complex fistulas, especially when an underlying Crohn’s disease is suspected [14], while others report an undisputed superiority of MRI [15]. However, these imaging techniques have some downsides: access to emergency MRI is often limited and it requires a long acquisition time, while EUS requires special skills and its use in an awake patient in the emergency setting is almost always precluded by intense anal pain. In this scenario, the use of CT scan offers multiple advantages, such as its short acquisition time and widespread availability. The main factor limiting CT’s accuracy is its poor spatial resolution in the pelvis and the difficulty to differentiate between a fistula tract and inflammation, because the tissue characteristics are very similar [16]. An old, small prospective trial found that CT scan was far less accurate than EUS in detecting fistulae, while the two methods were equivalent in the diagnosis of anorectal abscesses [17]. A retrospective study on 113 patients with anorectal abscesses found a 77% overall sensitivity of CT in detecting perirectal abscess, and this lack of sensitivity reduced further in immunocompromised patients [18]. Furthermore, some authors suggest that CT fistulography, with its accuracy rate of 81.1%, could be a helpful tool in selecting the most appropriate surgical treatment [19], but the population included in this prospective study is too small to make recommendations and does not relate to emergency presentations. The use of point-of-care trans-perineal ultrasound is emerging as a valid tool to diagnose perineal and perirectal abscesses in the emergency department. The advantages of this technique are multiple, but the results are highly operator sensitive due to the complex anatomy of the involved region and the substantial variability in patient presentation and location of rectal abscesses. In recent review, sonography demonstrated high accuracy in the identification of perianal abscesses [20], but the available evidence is too scarce to make a recommendation.

In conclusion, CT, MRI, and EUS could have a role in the diagnosis of perianal abscesses, with the principal aims of excluding related conditions and correctly determining the regional anatomy and extent of the disease; the choice of the appropriate imaging investigation should take into account multiple factors, such as patient’s past medical history, clinical presentation, local availability of resources, and skills. After the resolution of the acute phase, routine imaging after incision and drainage of the anorectal abscess is usually not required. An imaging follow-up is otherwise suggested in cases of recurrence, in the presence of suspected IBD and when there is evidence of a fistula and/or non-healing wound.

- **1.C - In patients with an anorectal abscess, what are the indications for surgical treatment and what is the appropriate timing for surgery?**

*In patients with anorectal abscess, we recommend a surgical approach with incision and drainage (strong recommendation based on low-quality evidence, 1C).*

*In patients with anorectal abscess, we suggest to base the timing of surgery on the presence and severity of sepsis (weak recommendation based on low-quality evidence, 2C).*

*In fit, immunocompetent patients with a small perianal abscess and without systemic signs of sepsis we suggest considering an outpatient management (weak recommendation based on low-quality evidence, 2C).*

The primary treatment of anorectal abscesses remains surgical drainage, with the timing being dictated by the severity and nature of any sepsis. In general, the incision should be kept as close as possible to the anal verge to minimize the length of a potential fistula, while still providing adequate drainage and avoiding sphincteral damage [21]. It is important to stress the high rate of recurrence after drainage, which can be as high as 44% [22, 23]. The risk factors associated with recurrence are inadequate drainage, loculations, horseshoe-type abscess, and time from disease onset to incision [23, 24]. The high recurrence rate and the associated risk factors emphasize the need for a complete and accurate drainage of the abscess. A recent randomized prospective study on needle aspiration treatment vs. incision and drainage of acute simple perianal abscess emphasized the importance of a complete drainage of the perianal abscess, with recurrence rates of 41% following needle aspiration compared to 15% after incision and drainage [25].

Young, fit patients without any signs of sepsis may have their surgery undertaken in an ambulatory setting, and small simple perianal abscesses may be treated under local anesthesia. While perianal and ischioanal abscesses should be treated via incision and drainage of the overlying skin, intersphincteric abscess should be drained into the rectal lumen and a limited internal sphincterotomy may be required [26]. Supralevator abscess may require drainage via the rectal lumen (if extension of an intersphincteric abscess) or externally via the skin (if extension of ischioanal abscess) [27, 28].

The timing for surgery is dictated by the patient’s clinical condition and comorbidities: the presence of sepsis, severe sepsis or septic shock, immunosuppression, diabetes mellitus, and diffuse cellulitis should prompt an emergent drainage. In the absence of these factors, the surgical drainage should ideally be performed within 24 h.

- **1.D - in patients with an anorectal abscess, what is the role of wound packing after surgical drainage?**

*No recommendation can be made regarding the use of packing after drainage of an anorectal abscess, based on the available literature.*

Common practice is to place an internal dressing (pack) into the cavity following incision and drainage of an anorectal abscess, both for hemostasis and to prevent the premature closure of the skin; the pack is then changed regularly until the cavity heals. Some authors suggest placing a catheter or drain into the abscess cavity, which drains into an external dressing, with a small stab incision under local anesthetic and to leave it in place until it stops draining.

Despite being commonly performed, the role of wound packing after anorectal abscess drainage remains unproven. Those supporting its use suggest a reduced time to healing and a reduced incidence of recurrence, but patients may experience pain during the process, and there is additional healthcare expense with its prolonged use. A recent Cochrane review [29] included two studies for a total of 64 randomized participants, and both included studies were at high risk for bias. For these reasons, the authors stated that it “is unclear whether using internal dressings (packing) for the healing of perianal abscess cavities influences time to healing, wound pain, development of fistulae, abscess recurrence, or other outcomes” and, as such, its use should be left to individual unit policy and patient discussion. A subsequent multi-center observational study was performed, enrolling 141 patients undergoing incision of primary anorectal abscess and subsequent packing. The conclusions of the authors were that packing is costly and painful and does not add benefit to the healing process [30]. Hopefully, an on-going randomized trial in the UK (PPAC2) may shed light on this in due course.

- **1.E - in patients with an anorectal abscess with a concomitant fistula, what are the indications for fistula treatment in the acute setting?**

*In patients with anorectal abscess and an obvious fistula, we suggest to perform a fistulotomy at the time of abscess drainage only in cases of low fistula not involving sphincter muscle (i.e., subcutaneous fistula) (weak recommendation based on low-quality evidence, 2C).*

*In patients with anorectal abscess and an obvious fistula involving any sphincter muscle, we suggest to place a loose draining seton (weak recommendation based on low-quality evidence, 2C).*

*In patients with anorectal abscess and no obvious fistula, we suggest against probing to search for a possible fistula, to avoid iatrogenic complications (weak recommendation based on low-quality evidence, 2C).*

A third of perianal abscesses may manifest a fistula-in-ano which increases the risk of abscess recurrence requiring repeat surgical drainage [31]. The edema and anatomical distortion that may be present with acute infection imply that the treating surgeon should be very cautious approaching an associated anal fistula. If an obvious fistula is found at the time of anorectal abscess drainage, this may only be laid open if deemed to be subcutaneous by an experienced surgeon, as treatment of an anal fistula at the time of abscess drainage is associated with a lower rate of recurrent abscess/fistula and need for further surgery. This has been confirmed by a prospective study by Schouten et al. [32], which found that primary fistulectomy with partial internal sphincterectomy performed at the time of abscess drainage highly reduced the recurrence rate, but was also associated with increased anal function disturbances; the authors concluded that fistulectomy should thus be reserved as a second stage procedure, if necessary. A 2010 Cochrane review [33] included six trials comparing incision and drainage of perianal abscess alone versus incision and drainage with fistula treatment, for a total of 479 patients. Their meta-analysis showed a significant reduction in recurrence, persistent abscess/fistula, or repeat surgery in favor of fistula surgery at the time of abscess incision and drainage, but this result is burdened by an increased, albeit statistically insignificant, incidence of continence disturbances at 1-year follow-up. According to these results, no attempt should be made to probe or use hydrogen peroxide to search for a possible fistula, in order to avoid iatrogenic complications. If there is suspicion of any sphincteric muscle involvement, then a loose draining seton [32, 34, 35] should be placed and subsequent management options should be discussed with the patient to avoid the risk of incontinence.

- **1.F - in patients with an anorectal abscess, is there a role for antibiotic therapy and what is the appropriate antibiotic regimen?**

*In patients with drained anorectal abscess, we suggest antibiotics administration in the presence of sepsis and/or surrounding soft tissue infection or in case of disturbances of the immune response (weak recommendation based on low-quality evidence, 2C).*

*In patients with anorectal abscess, we suggest sampling of drained pus in high-risk patients and/or in the presence of risk factors for multidrug-resistant organism infection (weak recommendation based on very low-quality evidence, 2D).*

A recent meta-analysis tried to shed light on the role of antibiotics after drainage of anorectal abscess. The meta-analysis included six studies for a total of 817 patients, of which 358 (43.8%) underwent management without antibiotics while 459 (56.2%) patients were treated with antibiotics: the fistula rate in subjects receiving antibiotics was 16% versus 24% in those not receiving postoperative antibiotics, with a 36% lower odds of fistula formation [36]. The authors concluded that an empiric 5–10 day course of antibiotics following operative drainage may reduce the incidence of post-operative fistula, but the evidence is low and they do not specify duration and type of antibiotics. One of the included studies retrospectively analyzed 172 patients with uncomplicated anorectal abscess and compared the outcomes of incision and drainage alone with incision and drainage plus 5 to 7 days of oral antibiotic therapy: they interestingly found that among patients with anorectal abscess complicated by surrounding cellulitis, induration, or systemic sepsis and treated with drainage alone, there was a 2-fold increase in recurrent abscess [36]. Conversely, the role of antibiotics is pivotal in neutropenic or otherwise immunosuppressed patients [37–41]. Furthermore, 2007 guidelines from the American Heart Association recommend administration of antibiotics before incision and drainage of the infected tissue in patients with prosthetic valves, previous bacterial endocarditis, congenital heart disease, and heart transplant recipients with valve pathology [42], while the role of prophylaxis in patients with bicuspid aortic valves and mitral valve prolapse remains debated [43].

Although routine cultures of drained pus are usually considered unnecessary [44], there are studies that highlight how the prevalence of methicillin-resistant *Staphylococcus aureus* (MRSA) in routine anorectal abscesses can be as high as 35% [45, 46]. For this reason, we suggest to consider sampling of drained pus in cases with risk factors for multidrug-resistant organism (MDRO) infection, in case of recurrent infections or non-healing wounds and in high-risk patients (e.g., HIV, immunocompromised patients, etc.) [44–47].

We refer to the WSES guidelines for soft-tissue [48] and intra-abdominal infections [49] for a discussion of the appropriate antibiotics regimens.

2. Perineal necrotizing fasciitis (Fournier’s gangrene)
  **2.A - in patients with suspected Fournier’s gangrene, what is the role of clinical examination and biochemical investigations?**

*In patients with suspected Fournier’s gangrene, we suggest to collect a focused medical history and a complete physical examination, including a digital rectal examination (weak recommendation based on low-quality evidence, 2C).*

*In patients with suspected Fournier’s gangrene and signs of systemic infection or sepsis, we suggest to request complete blood count and the dosage of serum creatinine and electrolytes, inflammatory markers (e.g., C-reactive protein, procalcitonin) and blood gas analysis, to assess the status of the patient (weak recommendation based on low-quality evidence, 2C). We also recommend to check serum glucose, hemoglobin a1c, and urine ketones to investigate an undetected diabetes mellitus (strong recommendation based on low-quality evidence, 1C).*

*In patients with suspected Fournier’s gangrene, we suggest to use Laboratory Risk Indicator for Necrotising Fasciitis (LRINEC) score for an early diagnosis and Fournier’s Gangrene Severity Index (FGSI) for prognosis and risk stratification (weak recommendation based on moderate quality evidence, 2B).*

Perineal necrotizing fasciitis, also called Fournier’s gangrene, is a rare and potentially life-threatening necrotizing infection involving the fascia and subcutaneous tissues of the external genitalia or perineum [50], with potential manifestations that may include septic shock and multiple organ failure. Debate on the precise definition of this condition is still ongoing, but the most commonly accepted was proposed by Smith et al. As “an infective necrotizing fasciitis of the perineal, genital, or perianal region” [51]. A large population-based epidemiological study from the USA [52] found an overall incidence of 1.6 Fournier’s gangrene cases per 100,000 males annually, accounting for less than 0.02% of all hospital admissions. Males are predominantly affected, with a male to female ratio of 42:1. The mean age of presentation is 51 years. Mortality rates reach 88% in some studies, but Sorensen and colleagues report a mortality rate of 7.5% [52]. Patients affected tend to have comorbidities, with diabetes and obesity the most frequently associated pathologies, but all those conditions that result in an impaired host resistance from reduced cellular immunity (i.e., alcoholism, HIV, leukemia) are associated with increased risk of Fournier’s gangrene [53]. According to the original description, the etiology was idiopathic, but the increased knowledge of this disease has identified three possible sites of origin of the infection: the perineal skin (24% of the cases), the colorectal region (21%), and the genito-urinary tract (19%); an unknown origin still remains for the remaining 36% of the cases [53]. The pathophysiology of the fasciitis starts with the presence of a localized infection that allows the entrance of normally commensal bacteria into the perineum (the infection is typically polymicrobial, with *Streptococcus*, *Staphylococcus*, and *Escherichia coli* commonly present); the subsequent inflammatory response results in an obliterative endarteritis, with thrombosis of the surrounding vessels and important reduction in blood flow to this region. The following tissue ischemia promotes further anerobic bacteria proliferation and fascial necrosis and digestion [54]. Diagnosis is mainly clinical and a focused and detailed medical history as well as a complete physical examination including a careful inspection of the perineum is mandatory. The most common symptoms of Fournier’s gangrene include perineal and/or scrotal pain, swelling, and erythema. Pain is usually intense even in the presence of scarce clinical findings at the perineal examination. Systemic features such as fever and tachycardia are often present. The examination may reveal purulent discharge, crepitus (subcutaneous emphysema), and patches of the necrotic tissue with surrounding edema. Cutaneous manifestations tend to appear later in the disease process as these patches progress to florid gangrene [55]. The infection rapidly spreads cranially to the abdominal wall and caudally to the legs, following the course of the superficial perineal fascia that is in continuity with Colle’s fascia and Scarpa’s fascia. Interestingly, testicular involvement is rare and this has been attributed to their non-perineal blood supply [54].

The tendency to spread widely and extremely quickly makes Fournier’s Gangrene a time-sensitive disease: prompt recognition and treatment are of utmost importance but are not always easy. Several risk scores have been proposed and the most commonly used are the Laboratory Risk Indicator for Necrotising Fasciitis (LRINEC) [56], the Fournier’s Gangrene Severity Index (FGSI) [57], and its simplified version (SFGSI) [58]. These scores seem to be reliable tools for diagnosis and prognosis in Fournier’s gangrene, even if their accuracy is still a matter of debate.

Regarding the LRINEC, some recent meta-analyses reach discordant results. The first by Bechar et al. [59] found a statistically positive correlation between LRINEC score and diagnosis of necrotizing fasciitis, defining the LRINEC score as a useful tool in the diagnosis and surgical treatment of patients with necrotizing fasciitis. In 2019, Fernando et al. [60] concluded that, given its poor sensitivity, LRINEC score should not be used to rule out a necrotizing soft-tissue infection and that in case of high clinical suspicion an early surgical consultation is required. In the same year, a third meta-analysis [61] found a variable range of sensitivity (43.2–80%), positive predictive value (57–64%), and negative predictive value (42–86%)” for the LRINEC and that patients with higher SOFA score, prolonged ICU and hospital stay, and mortality usually presents with higher LRINEC scores. More importantly, this meta-analysis demonstrated the LRINEC score is not sensitive in immunocompromised patients. In conclusion, these studies seem to agree that LRINEC should only be used to confirm the diagnosis of necrotizing fasciitis and not to exclude it, due to its poor sensitivity. Confirmation of the poor sensitivity of LRINEC score comes from a recent study by Cribb et al. [62]: this retrospective study included 138 patients with necrotizing fasciitis and found a 60% sensitivity for the LRINEC score for differentiating between cases of necrotizing fasciitis from controls with severe cellulitis. The authors then proposed a novel scoring system, the SIARI score (site other than lower limb, immunosuppression, age < 60 years, renal impairment, inflammatory markers) and stated that the SIARI score demonstrated superior discriminative ability compared with the LRINEC score in both the developmental and validation cohorts. However, this scoring system has not been validated in other studies yet.

FGSI score was developed in 1995 by Laor et al. [57] specifically for the evaluation of Fournier’s gangrene. FGSI includes nine clinical/laboratory parameters (i.e., temperature, heart rate, respiration rate, serum sodium, serum potassium, serum creatinine, hematocrit, white blood count, serum bicarbonate) and remains the most commonly used scoring system to predict patient mortality, with a reported sensitivity of 65–88% and specificity of 70–100% [63–66]. Most of the studies agree with the cutoff value set by Laor et al. as patients with a FGSI >9 have a 75% probability of death [57, 63, 65]. The debate on which scoring system performs best is still ongoing and contrasting results on FGSI’s reliability can be found in the literature [67, 68]. In 2010, Yilmazlar et al. proposed a modification, adding age and extent of disease to the FGSI [69]: the novel scoring system, called Uludag FGSI (UFGSI), was reported to be superior to FGSI in predicting patient mortality in one study, but not in another [66]. Later on in 2014, Lin et al. analyzed 84 patients and compared the parameters of FGSI between survivors and non-survivors. Based on the results of this analysis, they generated a simplified FGSI (SFGSI), including only the parameters that showed a significant difference between the two groups (serum creatinine, hematocrit, and serum potassium levels) [58]. The resulting scoring system provided sensitivity and specificity of 87% and 77% in predicting patient mortality, respectively. This scoring system has been validated in other studies and showed a good reliability for risk stratification [70].

In conclusion, there is still no agreement on which scoring system should be adopted but, based on the available literature, we can state that early diagnosis using LRINEC score and stratification of patients into risk categories using FGSI score (or its modified versions SFGSI and UFGSI) may help in guiding correct management.

Regarding laboratory tests, in the light of the above information, we suggest to start the diagnostic workup of every patient with suspected FG with complete blood cell count; serum sodium, potassium, glucose, creatinine, and magnesium; urea; inflammatory markers (e.g., C-reactive protein, procalcitonin); and coagulation assessment and lactate.

- **2.B - in patients with suspected Fournier’s gangrene which are the appropriate imaging investigations?**

*In stable patients with suspected Fournier’s gangrene, we suggest to consider performing a CT scan (weak recommendation based on low-quality evidence, 2C).*

*In patients with Fournier’s gangrene, we recommend that imaging should not delay surgical intervention (strong recommendation based on moderate quality evidence, 1B).*

*In patients with Fournier’s gangrene and hemodynamic instability persisting after proper resuscitation, we suggest against CT imaging (weak recommendation based on low-quality evidence, 2C).*

Diagnosis of Fournier’s gangrene is mainly clinical. Plain radiographs, ultrasonography, computed tomography (CT), and magnetic resonance imaging (MRI) may demonstrate gas in the soft tissue planes as well as help determine the extent of the disease [71]. Evidence of gas formation is present in nearly half of all patients with FG and is highly specific (94%). Ultrasonographic findings typically include marked thickening of the scrotal skin, soft tissue inflammation, collections/abscesses, and subcutaneous gas [72]. In addition, US can demonstrate paratesticular fluid, which is seen prior to clinical crepitus, and scrotal contents along with Doppler blood flow can be examined [73]. As imaging evaluation in patients with FG may be limited by several factors, including the presence of concurrent acute renal failure or patient hemodynamic instability, making transport to the imaging department unsafe, bedside and point-of-care ultrasound have emerged as useful tools for FG diagnosis [74, 75]. US has multiple advantages: it can be promptly performed at the patient’s bedside, can evaluate the scrotal contents, and does not require radiation or intravenous contrast [72]. For these reasons, US can be suggested in cases where a CT scan is not available or not feasible. Otherwise, a contrast-enhanced CT-scan plays an important role in diagnosing and planning the treatment of Fournier’s gangrene, because it has greater specificity for evaluating disease extent than plain radiography, US, or physical examination alone and may also identify a potential underlying cause [76]. CT has a sensitivity approaching 90% for the diagnosis of necrotizing soft tissue infections, in addition to high specificity (93.3%) [75, 77]. MRI with gadolinium contrast is an excellent soft tissue imaging modality, but is of limited value in an emergency setting due to its extended time of examination and limited access to emergency MRI scanning in many centers [75]. Furthermore, given the utmost importance of a timely surgical treatment, imaging is not mandatory in treating emergent cases of Fournier’s gangrene with clinical or hemodynamical impairment and we suggest not delaying surgical treatment in these patients to obtain any form of imaging.

- **2.C - in patients with Fournier’s gangrene, what are the indications for surgical treatment and what is the appropriate timing for surgery?**

*In patients with Fournier’s gangrene, we recommend surgical intervention as soon as possible (strong recommendation based on low-quality evidence, 1C).*

*In patients with Fournier’s gangrene, we suggest planning repeat surgical revisions (exploration and debridement) according to patient conditions (weak recommendation based on low-quality evidence, 2C).*

*In patients with Fournier’s gangrene, we suggest seriated surgical revisions until the patient is free of necrotic tissue (weak recommendation based on low-quality evidence, 2C).*

Fournier’s gangrene is a rapidly spreading disease with a high mortality rate, especially in cases of delayed treatment. Cornerstones of treatment are represented by antibiotic therapy, appropriate hemodynamic resuscitation, and emergent surgical therapy, with drainage of fluid collection and complete debridement of necrotic tissue. It has been shown that early and aggressive surgical debridement improves survival and reduces the number of surgical revisions [78–83]. Thus, surgical intervention should be performed as soon as possible in the presence of high suspicion for Fournier’s gangrene and subsequent surgical revisions should be planned based on the patient conditions (ideally every 12–24 h). Surgical revisions should be continued until the patient is free of necrotic tissue [48].

- **2.D - in patients with Fournier’s gangrene, what is the appropriate surgical approach?**

*In patients with Fournier’s gangrene, we suggest to remove all the necrotic tissue (weak recommendation based on low-quality evidence, 2C).*

*In patients with Fournier’s gangrene, we suggest a multidisciplinary and tailored approach based upon the extent of perineal involvement, the degree of fecal contamination, and the possible presence of sphincter or urethral damage (weak recommendation based on low-quality evidence, 2C).*

*In patients with Fournier’s gangrene, we suggest to perform orchiectomy or other genital surgery only if strictly necessary and possibly based on a urologic consultation (weak recommendation based on low-quality evidence, 2C).*

*In patients with Fournier’s gangrene, we suggest planning the surgical management of early and delayed surgical sequelae with a multidisciplinary and skilled team (weak recommendation based on low-quality evidence, 2C).*

Radical surgery, with complete removal of all visible necrotic tissue, may be sufficient to treat the infection. In most severe cases, with deep tissue involvement, orchiectomy, colostomy, and percutaneous suprapubic cystostomy may be considered. Common indications for colostomy are anal sphincter involvement, fecal incontinence, and continued fecal contamination of the wound [73]. Fecal diversion via colostomy is obviously beneficial for wound healing, but carries a potential burden of morbidity (and usually requires a subsequent surgical procedure for stoma closure). Furthermore, a retrospective study by Ozturk et al. shows that temporary stoma formation significantly increases healthcare costs without affecting mortality rates and hospital length of stay [84]. These authors also suggest to postpone the decision regarding stoma creation for at least a 48-h period of observation from the initial surgery, to allow acute inflammation and edema regression, thus enabling a correct evaluation of sphincters and perianal tissues. In the light of the above, a multidisciplinary approach is needed, with early involvement of general or emergency surgeons, urologists and intensivists, and plastic surgeons where available [85]. Alternatively to colostomy, temporary fecal management system has been introduced for fecal diversion [86], but its use should be limited to short periods of time to avoid intra-rectal damage due to the device itself. Regarding urinary diversion, common indications for suprapubic urinary diversion include extensive penile and perineal debridement, urethral involvement, and periurethral abscesses [87], although most suggest that urinary catheterization provides satisfactory diversion [88] utilizing suprapubic cystotomy only in patients experiencing urethral disruption or stricture [89]. In conclusion, the decision regarding the need for fecal or urinary diversion and the best way to achieve them should be made by a multidisciplinary team and should be tailored to the characteristics of the individual patient.

Negative pressure wound therapy (NPWT) plays an important role in managing soft tissue infections [90]. NPWT has multiple advantages when compared to traditional dressings: it increases blood supply and encourages migration of inflammatory cells into the wound region; removes exudate, bacteria, and their products; and promotes the formation of granulation tissue [86]. The main downside of NPWT for the management of FG is the frequent involvement of the anal area that requires either a NPWT dressing change after every bowel movement or a bowel diversion system [91].

Due to the anatomical region involved, disfiguring surgery and sexual dysfunction may be unavoidable due to the necessarily aggressive surgery and may require subsequent complex genital reconstructive surgery and skin grafting [92, 93]. Reconstructive surgery goes beyond the aims of these guidelines, but the complexity of the reconstructive procedure and the psychological burden for the patient require a dedicated multidisciplinary team.

- **2.E - in patients with Fournier’s gangrene, which is the appropriate antibiotic regimen?**

*In patients with Fournier’s gangrene, we recommend starting an empiric antimicrobial therapy as soon as the diagnosis is suspected (strong recommendation based on moderate quality evidence, 1B).*

*In patients with Fournier’s gangrene, we recommend that empiric antimicrobial therapy should include cover for gram-positive, gram-negative, aerobic and anaerobic bacteria, and an anti-MRSA agent (strong recommendation based on moderate quality evidence, 1B).*

*In patients with Fournier’s gangrene, we recommend to obtain microbiological samples at the index operation (strong recommendation based on moderate quality evidence, 1B).*

*In patients with Fournier’s gangrene, we recommend to base antimicrobial de-escalation on clinical improvement, cultured pathogens, and results of rapid diagnostic tests where available (strong recommendation based on moderate quality evidence, 1B).*

### **Proposed regimens**

#### **In stable patients**

- *Piperacillin/tazobactam 4.5 g 6-hourly*
- +
- *Clindamycin 600 mg 6-hourly*

#### **In unstable patients**

- One of the following antibiotics
- *Piperacillin/tazobactam 4.5 g 6-hourly*
- *Meropenem 1 g 8-hourly*
- *Imipenem/Cilastatin500 mg 6-hourly*
- +
- One of the following antibiotics
- *Linezolid 600 mg 12-hourly*
- *Tedizolid 200 mg 24-hourly*

*Or*

- Another anti MRSA-antibiotic as
- *Vancomycin 25–30 mg/kg loading dose then 15–20 mg/kg/dose 8-hourly*
- *Teicoplanin loading dose 12 mg/kg 12-hourly for 3 doses, then 6 mg/kg 12-hourly*
- Daptomycin 6–8 mg/kg 24-hourly*
- *Televancin 10 mg/kg 24-hourly*
- +
- *Clindamycin 600 mg 6-hourly*
*Approved at the dosage of 4 mg/kg/24 h, it is currently used at higher dosages

Antibiotic treatment of FG should be prompt and aggressive, starting empiric antimicrobial therapy as soon as the diagnosis is considered. Initial coverage should be broad, including for gram-positive, gram-negative, aerobic and anaerobic organisms and, based on local epidemiology, an anti-methicillin-resistant *S. aureus* (MRSA) agent [94]. Microbiological samples must be sent at the index operation to obtain an antibiogram, thus allowing modification of the drug regimen based on the specific cultured pathogens. If inflammatory markers do not improve, it is mandatory to rule out an alternative or additional source of infection or a residual area of gangrene with further surgical exploration advised. Hyperbaric oxygen therapy may help in treating soft tissue infections [87, 95, 96]. For a detailed discussion of the management of skin and soft-tissue infections, we refer to the WSES/SIS-E guidelines [48].

3) Complicated hemorrhoids (thrombosed, strangulated, or bleeding)
  **3.A - in patients with suspected complicated hemorrhoids, what is the role of clinical examination and biochemical investigations?**

*No recommendation can be made regarding the role of biochemical investigations in patients with suspected thrombosed or strangulated hemorrhoids, based on the available literature.*

*In patients with suspected bleeding hemorrhoids, we suggest to collect a focused medical history and to perform a complete physical examination, including a digital rectal examination, to rule out other causes of lower gastrointestinal bleeding (weak recommendation based on low-quality evidence, 2C).*

*In patients with suspected bleeding hemorrhoids, we suggest to check vital signs, to determine hemoglobin and hematocrit, and to assess coagulation to evaluate the severity of the bleeding (weak recommendation based on low-quality evidence, 2C). In case of severe bleeding, we suggest blood typing and cross-matching (weak recommendation based on very low-quality evidence, 2D).*

The usual presentation of patients with complicated hemorrhoids is either acute anal pain (also called hemorrhoidal crisis) or anorectal bleeding. In both cases, a complete medical history, with a focus on the acute complaint, and a complete physical examination (comprehensive of digital rectal examination) are mandatory to rule out other possible causes of acute anal pain or bleeding. This division according to the main complaint at the ER referral will be used also for the following paragraphs on hemorrhoids. There is no literature regarding the role of biochemical investigations in case of a hemorrhoidal crisis: in this specific setting, the laboratory tests should be performed to exclude other causes of acute anal pain (i.e., anorectal abscesses, Fournier’s gangrene, anal fissures). It is easy to understand how important medical history and physical examination are in starting and driving the diagnostic process. On the other hand, patients referring with anorectal bleeding fall under the bigger category of acute lower gastrointestinal bleeding (LIGB) and should be investigated to exclude other causes of bleeding. In these cases, it is crucial to define the severity of bleeding and to correctly stratify the risk for every patient. Blood test should include a complete blood count (CBC), serum electrolytes, blood urea nitrogen (BUN), creatinine, and the coagulation assessment [97]. Blood type and crossmatch for possible transfusion of blood components should also be ordered at the time of initial assessment for patients with signs of severe bleeding. Multiple risk stratification scores are described in literature [98–104] and most of them include vital signs and hemodynamic parameters; presence of blood at rectal examination; laboratory tests, such as hematocrit, creatinine, and albumin; presence of comorbidities; and use of anticoagulant or antiplatelet drugs. In conclusion, the diagnostic workup of a patient presenting with hematochezia deriving from suspected bleeding hemorrhoids should start with the assessment of vital signs, followed by the collection of an accurate medical history and a thorough physical examination including digital rectal examination; then it should proceed with the execution of the blood tests necessary to determine the severity of the bleeding and to correctly stratify the risk for this patient. It is also advisable to carry out a pregnancy test with the woman’s consent if there is any doubt about whether she could be pregnant.

- **3.B - in patients with suspected complicated hemorrhoids, which are the appropriate imaging investigations?**

*In patients with suspected complicated hemorrhoids, we suggest to perform imaging investigation (CT scan, MRI, or endoanal ultrasound) only if there is suspicion of concomitant anorectal diseases (sepsis/abscess, inflammatory bowel disease, neoplasm) (weak recommendation based on low-quality evidence, 2C).*

The literature regarding complicated hemorrhoids is extremely scarce, and none of the studies investigate the role of imaging techniques in this specific subset of patients. Acute anal pain is the typical manifestation of multiple anal diseases, including hemorrhoidal crisis, anorectal abscesses, and anal fissures. The presence of an associated anorectal mass may further complicate the scenario, considering that not only thrombosed and prolapsed hemorrhoids could present as an anorectal mass, but also condylomas, abscesses, polyps, anorectal prolapse, or anorectal cancer can present in the same way [105]. Lastly, it is important to remember that acute complicated hemorrhoids could be the clinical manifestation of a concomitant inflammatory anal and perianal condition, as demonstrated by the reported incidence of symptomatic hemorrhoids in patients with inflammatory bowel disease (IBD) ranging from 3.3 to 20.7% [106]. Therefore, a thorough medical history and a complete physical examination are key factors to drive the diagnostic workup of acute anal pain. Imaging studies should be considered when there is suspicion of an underlying diseases. The only study investigating the role of EUS in hemorrhoidal disease is from Poen et al. [107] and concluded that hemorrhoids are associated with endosonographic thickening of submucosal tissue, internal and external anal sphincter, but EUS changes cannot predict treatment outcome or symptom recurrence. The conclusions of the abovementioned studies regarding imaging techniques in anorectal abscesses can be extended to complicated hemorrhoids workup, even if they were not aimed to analyze specifically the role of imaging in hemorrhoidal disease; however, the recommendations are weak.

- **3.C - in patients with complicated hemorrhoids, what is the role of endoscopy?**

*In patients with complicated hemorrhoids, we suggest to perform anoscopy as part of the physical examination, whenever feasible and well tolerated (low recommendation based on low-quality evidence, 2C).*

*In patients with complicated hemorrhoids, we suggest to perform colonoscopy in case of concern for inflammatory bowel disease or cancer arising from patient personal and family history, or from physical examination (low recommendation based on low quality evidence, 2C).*

Endoscopy has a pivotal role in the diagnosis and management of LGIB. Most authors recommend the use of anoscopy in all patients referred with complicated hemorrhoids [108–110], and this opinion is supported by multiple studies that highlight the good accuracy of anoscopy in detecting hemorrhoids and other anorectal lesions, when compared to flexible endoscopy [111, 112]. However, patients with trombosed and strangulated hemorrhoids usually experience excruciating anal pain and therefore it is not possible to perform an accurate anoscopy in an awake patient in this setting. Anoscopy could be an option for the evaluation of painless anorectal bleeding, while its application in case of hemorrhoidal crisis usually requires proper sedation. The next step is to determine which patients requires a full colonoscopy; it is extremely important not to blindly attribute painless rectal bleeding to hemorrhoids, because it may be a sign of other diseases (i.e., colorectal cancer, inflammatory bowel disease or other colitis, diverticular disease, angiodysplasia) [113]. According to the American Society of Colon and Rectal Surgeons, further endoscopic evaluation is warranted if there is concern for inflammatory bowel disease or cancer arising from a detailed patient personal and family history, and from a physical examination which may include anoscopy/proctoscopy and/or flexible sigmoidoscopy [109]. Obviously, risk factors include anemia, atypical bleeding, alteration of bowel habits, preceding weight loss, and personal and family history of colorectal cancer or IBD.

- **3.D - in patients with complicated hemorrhoids, which is the appropriate pharmacological regimen?**

*In patients with complicated hemorrhoids, we recommend non-operative management as the first-line therapy, with dietary and lifestyle changes (i.e., increased fiber and water intake together with adequate bathroom habits) (strong recommendation based on moderate quality evidence, 1B).*

*In patients with complicated hemorrhoids, we suggest to administer flavonoids to relieve symptoms (weak recommendation based on moderate quality evidence, 2B).*

*In patients with thrombosed or strangulated hemorrhoids, we suggest the use of topical muscle relaxant (weak recommendation based on low quality evidence, 2C).*

*No recommendation can be made regarding the role of NSAIDs, topical steroids, other topical agents, or injection of local anesthetics for complicated hemorrhoids, based on the available literature.*

Medical and pharmacological therapies are by far the most widely adopted therapy for complicated hemorrhoids and are supported by several studies. A Cochrane review from 2005 investigated the role of laxatives for the treatment of symptomatic hemorrhoids and included seven randomized trials enrolling a total of 378 participants to fiber or a non-fiber control [114]: meta-analyses using random-effects models showed that laxatives in the form of fiber had a beneficial effect in the treatment of symptomatic hemorrhoids, with the greatest effect in the reduction of bleeding. The authors warned however that the risk of publication bias and the moderate quality of the included studies may limit the strength of the results of the meta-analysis. Another meta-analysis from the same author focusing only on fiber intakes versus non-fiber controls found the same results [115].

The role of flavonoids and phlebotonics has been extensively studied. Phlebotonics are a heterogeneous class of drugs, consisting of plant extracts (i.e., flavonoids) and synthetic compounds (i.e., calcium dobesilate), mainly used to treat chronic venous insufficiency. Two major meta-analyses were performed, the first one in 2006 [116] and the second one, developed by the Cochrane Collaboration, in 2012 [117]. The latter included twenty-four randomized controlled trials: twenty of these studies (enrolling a total of 2344 participants) evaluated the use of phlebotonics versus a control intervention, two compared different forms of phlebotonics with each other, one study evaluated phlebotonics with a medical intervention, and one study compared the use of phlebotonics with infrared photocoagulation. Phlebotonics demonstrated a statistically significant beneficial effect for the outcomes of pruritus, bleeding, bleeding post-hemorrhoidectomy, discharge and leakage, and overall symptom improvement. On the other hand, the benefit for pain, pain scores post-hemorrhoidectomy, or post-operative analgesic consumption is present but did not reach statistical significance. Again, the definition of “symptomatic” hemorrhoids used in these studies is unclear and makes it difficult to determine whether the symptoms are acute or chronic; for these reasons, we cannot make strong recommendations for an acute care setting.

Severe anal pain is the most common symptom associated with acute thrombosed external hemorrhoids; internal anal sphincter hypertonicity is both deriving from and causing this excruciating pain. Furthermore, the spasm of anal sphincter worsens the congestion of the prolapsed piles. For these reasons, the use of muscle relaxant, like topical nitrates and calcium channels antagonists, has been proposed for the treatment of thrombosed hemorrhoids. The role of calcium antagonists was studied in 2001 in a prospective randomized study by Perrotti et al. [118] that enrolled 98 patients with thrombosed hemorrhoids: the study group (50 patients) received topical 0.3% nifedipine and 1.5% lidocaine ointment every 12 h for 2 weeks, while the control group (48 patients) received only topical 1.5% lidocaine ointment. The authors found a sharp increase in resolution rate of acute thrombosed external hemorrhoids after 14 days of therapy with topical Nifedipine (92% resolution rate in the study group as opposed to 45.8% in the control group), without observing any systemic side effect. The small number of enrolled patients obviously limits the power of this study, but the results are encouraging. The effects of topical nitrates were analyzed in another prospective study and showed good results, but these drugs are burdened by a high incidence of headache that may limit their use [119].

The role of topical anti-thrombotic is not yet clear. Only one study focused specifically on the use of topical anti-thrombotic therapy for acute hemorrhoids and heparin treatment was found to significantly improve healing and resolution of acute hemorrhoids [120, 121]. However, the small number of patients included in this study does not consent to make any recommendation regarding the role of this drugs in the treatment of complicated hemorrhoids.

Steroid cream should be applied for no more than 7 days, and a long-term use should be avoided because of potential thinning of perianal and anal mucosa and increasing risk of injury [122, 123]. There are no scientific data evaluating either NSAIDS, cortisone, and its derivates or injection of local anesthetics for the treatment of complicated hemorrhoids.

- **3.E - in patients with complicated hemorrhoids, what is the role of office-based procedures?**

*No recommendation can be made regarding the role of office-based procedures (i.e., rubber band ligation, sclerotherapy, infrared coagulation) in complicated hemorrhoids, based on the available literature.*

The literature regarding the utilization of office-based procedures (i.e., rubber band ligation, injection sclerotherapy, infrared coagulation, cryotherapy, radiofrequency ablation, laser therapy, etcetera) is significant but generally heterogeneous and of low quality: the number of published studies is enormous and the definition of bleeding or symptomatic hemorrhoids is always unclear. Regarding sclerotherapy, there are anecdotal reports of its application in patients with acutely bleeding hemorrhoids [124].

For these reasons, there is not enough quality data to make any recommendation regarding the application of office-based procedures to manage complicated hemorrhoids.

- **3.F - in patients with complicated hemorrhoids, what are the indications for surgical treatment and what is the appropriate timing for surgery?**

*In patients with thrombosed hemorrhoids, we suggest to base the decision between non-operative management and early surgical excision on local expertise and patient’s preference (weak recommendation based on low quality evidence, 2C).*

*In patients with thrombosed hemorrhoids, we suggest against the use of incision and drainage of the thrombus (weak recommendation based on low-quality evidence, 2C).*

*No recommendation can be made regarding the role of surgery in patients with bleeding hemorrhoids, based on the available literature.*

Despite the high incidence of complicated hemorrhoids among the general population, there are only few studies on the surgical treatment of thrombosed hemorrhoids and none on bleeding hemorrhoids. Furthermore, the definitions of “thrombosed hemorrhoids,” “hemorrhoidal crisis,” “prolapsed hemorrhoids,” and “bleeding hemorrhoids” are multiple and confusing, and most of the currently available studies focus on chronic complaints and elective procedures. The evidence is thus scarce and of low quality, making it extremely difficult to provide evidence-based recommendations.

Focusing on thrombosed hemorrhoids, the studies comparing non-operative management (NOM) and surgery led to contrasting results. A prospective study from Allan et al. [125] compared the outcome of urgent hemorrhoidectomy with conservative treatment for prolapsed thrombosed internal hemorrhoids and found that conservative treatment was associated with shorter patient stay and less anal sphincter damage compared with operative treatment. On the other hand, other studies suggest that surgery may be superior to conservative management, but the correct duration of NOM and the timing for surgery are not known, as confirmed by the literature research performed by Chan et al. [126]. Patients treated conservatively will eventually have a resolution of symptoms, but recurrence rate is high and there is some evidence that surgical excision may result in more rapid symptom resolution, lower incidence of recurrence, and longer remission intervals. Simple incision of thrombosed hemorrhoids with clot removal used to be a popular procedure but has been abandoned by most specialists because of persistent bleeding and the significantly higher recurrence rates seen. A randomized prospective study by Cavcić et al. [127] divided 150 patients with acutely thrombosed hemorrhoids into three different arms: topical application of 0.2% nitroglycerin, incision and evacuation of thrombus, and excision of the hemorrhoid; the analysis of pains scores on day 4 after treatment showed that thrombus evacuation provided the worst results. Another study from Greenspoon et al. [128] retrospectively reviewed 231 patients who underwent treatment for external hemorrhoid thrombosis from 1990 to 2002, of which 51.5% was managed conservatively and 48.5% underwent surgical treatment. Interestingly, they found a significantly shorter time to symptom resolution in patients treated surgically (3.9 days vs 24 days), as well as a reduced incidence of recurrence (6.3% vs 25.4%). In this study, patients were allowed to choose between excision or conservative therapy and the authors stated that “the decision to manage patients surgically reflects both patient and physician preference”. Jongen et al. [129] retrospectively analyzed 340 patients undergoing excision of thrombosed external hemorrhoids under local anesthesia between 1995 and 2000 and concluded that outpatient excision under local anesthesia can be safely performed with a low recurrence and complication rate. On the other hand, Ceulemans et al. compared results after elective and emergency hemorrhoidectomy and found that early complications, reoperation, and late anal stenosis were more common after emergency than elective hemorrhoidectomy [130]. Older work by Eu et al. confirm the increased rate of late anal stenosis after emergency hemorrhoidectomy, but found similar results in terms of recurrence and other complications [131]. A couple of other studies tried to answer the question of whether stapled hemorrhoidectomy was comparable to conventional hemorrhoidectomy for thrombosed hemorrhoids. These four studies are prospective and randomized but are based on small cohorts of patients (maximum 40 patients per arm) [132–135]; their conclusions are similar, reporting better results after stapled hemorrhoidectomy in terms of less postoperative pain, shorter operation time and hospital stay, and earlier return to normal activity.

In conclusion, based on the paucity of available studies and on the small numbers of patients included, we believe that hemorrhoidectomy can be beneficial in selected patients and the decision between NOM and early surgical excision should be based on physician’s expertise and patient’s preference; furthermore, we suggest against the use of incision and drainage of the thrombus, given the higher incidence of bleeding and relapse of symptoms. The evidence about stapled hemorrhoidectomy is scarce and, also given the potential for life-threatening complications of this procedure in the elective setting [136], we do not make any recommendation for its application in an acute care - emergency setting.

This literature review could not identify any data regarding the role of surgery for bleeding hemorrhoids, so we cannot make any recommendations in this setting.

- **3.G - in patients with complicated hemorrhoids, what is the role of angiography?**

*No recommendation can be made regarding the role of angiography in complicated hemorrhoids, based on the available literature.*

There is growing evidence that angiography and angioembolization could have a role for the treatment of hemorrhoids in an elective setting. The reported results are encouraging [137–140], but there are not enough data to make any recommendation regarding its application to an acute care - emergency setting.

4) Bleeding anorectal varices
  **4.A - in patients with suspected bleeding anorectal varices, what is the role of clinical examination and biochemical investigations?**

*In patients with suspected bleeding anorectal varices, we suggest to collect a focused medical history and to perform a complete physical examination, including a digital rectal examination, to rule out other causes of lower gastrointestinal bleeding (weak recommendation based on low-quality evidence, 2C).*

*In patients with suspected anorectal varices, we suggest to check vital signs, to determine hemoglobin and hematocrit, and to assess coagulation, to evaluate the severity of the bleeding (weak recommendation based on low-quality evidence, 2C). In case of severe bleeding, we suggest blood typing and cross-matching (weak recommendation based on very low-quality evidence, 2D).*

Anorectal varices (ARV) have been defined as discrete, dilated, and submucosal veins, extending proximal to the dentate line and into the rectum. They are a known complication of portal hypertension and can occur in up to 89% of patients with the portal pressure above 10 mmhg, although the overall incidence in the general population is low [141]. Despite this high prevalence, serious hemorrhage from ARV is uncommon, with significant bleeding reported in less than 5% of such patients, but can be fatal [141]. The presence of ARV is not directly related to a specific cause of portal hypertension, nor to the grade or extension of other varices (esophageal or gastric); the only clue to suspect this disease is the presence of anorectal bleeding in patients with a history of long-standing or uncontrolled portal hypertension.

As already mentioned in relation to bleeding hemorrhoids, the first assessment of patients with haematochezia should follow the indications for the management of acute LIGB. In the case of ARV, a correct and thorough medical history is crucial to suspect this specific disease and can guide the subsequent diagnostic process. It is necessary to define the severity of bleeding and to correctly stratify the risk for every patient. Blood test should include a CBC, serum electrolytes, blood urea nitrogen, creatinine, and the coagulation assessment [97]. Blood type and crossmatch for possible transfusion of blood components should also be ordered at the time of initial assessment for patients with signs of severe bleeding. Furthermore, it is advisable to carry out a pregnancy test with the woman’s consent if there is any doubt about pregnancy. The risk stratification strategy is superimposable to what has already been discussed in the bleeding hemorrhoids section.

- **4.B - in patients with suspected bleeding anorectal varices, which are the appropriate imaging investigations?**

*In patients with bleeding anorectal varices, we suggest EUS +/- color Doppler evaluation as a second line diagnostic tool, especially for deep rectal varices or when in doubt (weak recommendation based on low-quality evidence, 2C).*

*In patients with bleeding anorectal varices and failed detection of bleeding site at endoscopy and EUS, or whenever EUS is not available, we suggest to perform contrast-enhanced CT-scan (weak recommendation based on low-quality evidence, 2C).*

*In pregnant patients with bleeding anorectal varices and failed US detection of bleeding site, we suggest to perform MRI angiography, if available and if allowed by the clinical scenario (weak recommendation based on low-quality evidence, 2C).*

The diagnosis of ARV bleeding can be difficult, especially when bleeding is massive. Should the diagnosis remain open to doubt, even after endoscopy, supplementary diagnostic procedures can be considered and endoscopic ultrasound (EUS) can be of great help: multiple studies showed that EUS can detect deep rectal varices in a large proportion of patients who do not have identified varices on routine endoscopy [142] and that EUS is better than endoscopy in detecting rectal varices (85% vs 45%) and in determining their number [143]. Furthermore, a series of studies from Sato and colleagues showed a great benefit with the implementation of EUS with color Doppler evaluation: in fact, the color doppler examination shows precisely the anatomy of the entire rectal venous plexus (intramural rectal varices, perirectal collateral veins, and communicating veins between intramural rectal varices) through color flow images and allows the evaluation of the hemodynamics of varices calculating the velocity of blood flow in rectal varices; this latter evaluation helpful in identifying high-risk group for rectal variceal rupture (the fastest the blood flow is, the more likely the ARV will bleed) [144–146].

In case both endoscopy and EUS fail to identify the bleeding, other imaging investigation could be useful. Barium enema is now only historical. MRI-venography is also helpful in assessing patients both pre- and post-transjugular intrahepatic portosystemic shunt (TIPS) and in diagnosing other sites of abnormal varices [147]. MRI has multiple downsides, making it at least problematic to perform in an emergency setting: it is not available in every facility and at every hour and it is a lengthy investigation in terms of time. For these reasons, CT scan is a better option in an acute care setting. In 2012, Moubarak et al. [148] provided a detailed description of all the possible portosystemic collateral vessels that can be found in patients with liver cirrhosis using a three-dimensional contrast-enhanced CT-scan. Although there are still limited data in the literature, it is thought that CT may be able to detect bleeding rates as low as 0.35 ml/min. The overall sensitivity of CT for acute hemorrhage may be as high as 92% and, unlike other diagnostic modalities, even in cases where a site of active extravasation is not identified, CT can potentially identify other findings to suggest a cause or site of origin for the bleeding [149]. CT has the advantage of a very quick acquisition but, given that bleeding from rectal varices is not truly arterial in nature, active extravasation is almost never visualized. Nevertheless, the visualization of large serpiginous veins both surrounding the rectum (pararectal varices) and within the rectal wall itself (rectal varices) on portal venous phase images can be highly suggestive; CT has also the ability to rule out other sources of bleeding and may provide information on the underlying etiologies of vascular and nonvascular diseases, such as inflammatory or neoplastic lesions. A retrospective study from Nagata et al. [150] comprised 223 patients emergently hospitalized for LGIB who underwent early colonoscopy within 24 h from arrival at the hospital and compared the bleeding source rate detection between two strategies: early colonoscopy following urgent CT or early colonoscopy alone. They interestingly found that the detection rate was higher with colonoscopy following CT for vascular lesions leading to more endoscopic therapies and concluded that urgent CT before colonoscopy had about 15% additional value for detecting vascular lesions compared to colonoscopy alone.

- **4.C - in patients with suspected bleeding anorectal varices, what is the role of endoscopy?**

*In patients with suspected bleeding anorectal varices, we suggest the use of ano-proctoscopy or flexible sigmoidoscopy as the first-line diagnostic tool (weak recommendation based on low-quality evidence, 2C).*

*In patients with suspected bleeding anorectal varices and high-risk features or evidence of ongoing bleeding, we suggest to perform an urgent colonoscopy (plus upper endoscopy) within 24 h of presentation (weak recommendation based on low-quality evidence, 2C).*

*In patients with suspected bleeding anorectal varices and risk factors for colorectal cancer or suspicion of a concomitant more proximal source of bleeding, we suggest to perform a full colonoscopy (weak recommendation based on low-quality evidence, 2C).*

*In patients with bleeding anorectal varices, we suggest to use local procedures, such as endoscopic variceal ligation, endoscopic band ligation, sclerotherapy or EUS-guided glue injection, to arrest bleeding in first instance where feasible (weak recommendation based on low-quality evidence, 2C).*

It is extremely important to make a correct differential diagnosis between hemorrhoids and ARV, but this distinction can be difficult, especially in case of massive bleeding. Occasionally, patients may have other causes of massive bleeding, e.g., solitary rectal ulcer syndrome or diverticulosis of colon. Thus, a correct diagnosis is essential. At clinical examination with proctoscope or anoscope usually ARV are discrete, compressible and serpiginous submucosal varicose veins that cross the dentate line and extend cranially into the rectum, while hemorrhoids are abnormal anal cushions with dilatation of hemorrhoidal venous plexus that are confined within the anal canal. Thus, simple clinical examination using a proctoscope or an anoscope is a valuable method for accurately identifying ARV and some studies have shown that flexible sigmoidoscopy and colonoscopy are as effective as anoscopy in diagnosing ARV [151–153]. It is important to remember that patients with ARV might have other, more proximal, sources of bleeding and anorectal bleeding presents a clinical dilemma in terms of associated risk of colorectal cancer (CRC). The estimated risk of CRC in patients with rectal bleeding has been reported to range from 2.4 to 11%, hence a full colonoscopy may be necessary to identify such a source [141]. Furthermore, a randomized trial from Laine et al. [154] showed that up to 15% of patients presenting with serious hematochezia (defined as alteration of hemodynamic status, hemoglobin drop ≥1.5g/dl, or necessity of blood transfusion) have an upper gastrointestinal source of bleeding identified at upper endoscopy. Another interesting study by Jensen et al. [155] found that even in a selected cohort of patients with diverticulosis and hematochezia, up to 8% of bleedings have an upper source. We suggest to perform an upper endoscopy in all cases where a clear diagnosis of bleeding ARV is not possible.

Endoscopy also has a therapeutic role in these patients and those with high-risk features or who have evidence of ongoing bleeding should undergo an urgent colonoscopy within 24 h of presentation [156]. The use of local treatment is described in the literature for both controlling acute bleeding from ARV and in the secondary prevention of bleeding, but there is no evidence supporting a prophylactic treatment of asymptomatic ARV. Multiple techniques have been reported [157–169], but a thorough discussion of the endoscopic approach goes beyond the aims of this guidelines.

- **4.D - in patients with bleeding anorectal varices, what is the role of non-operative management?**

*In patients with bleeding anorectal varices, we suggest multidisciplinary management, early involving the hepatology specialist team and focusing on optimal control of comorbid conditions (weak recommendation based on very low-quality evidence, 2D).*

*In patients with anorectal varices and mild bleeding, we suggest intravenous fluid replacement, blood transfusion if necessary, correction of coagulopathy, and optimal medication for portal hypertension (weak recommendation based on low-quality evidence, 2C).*

*In patients with anorectal varices and severe bleeding, we recommend to maintain an Hb level of at least > 7 g/dl (4.5 mmol/l) during the resuscitation phase and a mean arterial pressure > 65 mmhg, but avoiding fluid overload (strong recommendation based on moderate-quality evidence, 1B).*

*In patients with bleeding anorectal varices, we suggest the endorectal placement of a compression tube as a bridging maneuver, to help stabilization of the patient or to allow the transfer to a tertiary hospital (weak recommendation based on very low-quality evidence, 2D).*

The management of bleeding ARV can be very challenging and essentially includes prompt resuscitation and correction of coagulopathy, immediate workup to localize the site/source of bleeding and then the application of a suitable treatment modality or immediate transfer to a tertiary referral center. Management options may vary with a combination of local/endoscopic, medical, radiological, and surgical methods available. In mild cases, intravenous fluid replacement, blood transfusion, correction of coagulopathy, and optimal medication for portal hypertension are usually effective.

Initial resuscitation and hemodynamic stabilization are critical and patients’ conditions should be optimized before endoscopic intervention. The management of comorbid conditions, including the appropriate management of anti-platelet agents and anticoagulants, requires a multidisciplinary and individualized approach.

The intravascular volume repletion is done with crystalloids and packed red blood cells. The targets of resuscitation are the restoration of hemodynamic status but avoiding over-expansion, which may exacerbate portal pressure, impair clot formation, and increase the risk of further bleeding [170]. In fact, a certain degree of hypovolemia and hypotension promotes activation of endogenous vasoactive systems, leading to splanchnic vasoconstriction and, therefore, reducing portal blood flow and pressure. A recent randomized controlled trial showed that a restrictive packed red blood cell transfusion strategy improves survival in Child–Pugh A and B patients. The results of this study showed that patients with cirrhosis and acute variceal bleeding should be transfused when hemoglobin drops below 7 g/dl, aiming at a target level of 7–9 g/dl [171]. Other risks related to blood product utilization are immunologic (transfusion-related lung injury and development of HLA antibodies) and can impact subsequent transplantation or impair the ability to receive further transfusions [172–174]. A recent guideline from the American Gastroenterology Association (AGA) Institute Clinical Practice Updates Committee [175] suggests the following transfusion thresholds for management of active bleeding in advanced liver disease: hematocrit >25%, platelet count >50,000, and fibrinogen >120 mg/dl. Exceptions to this restrictive strategy are massive bleeding, which should prompt the activation of a dedicated transfusional protocol, cardiovascular comorbidities, and any other condition that precludes adequate physiological response to acute anemia. It is important to keep in mind that an acute hypotension may decrease hepatic perfusion, which in the setting of underlying chronic liver disease can exacerbate liver injury. A helpful maneuver to slow down the bleeding rate could be the endorectal placement of a compression tube (i.e., Sengstaken-Blakemore Tube or a Linton-Nachlas balloon compression tube) [141, 176–181] held under moderate traction: this device should be left in place until the patient is sufficiently stabilized or until the patient is transferred to a center with available expertise. The placement of balloon tamponade can also give additional time to correctly study the patients with CT scans or other imaging techniques, thus enabling to tailor the management strategy to the anatomy of the bleeding vessel [182]. Eventually, rebleeding is almost universal if another modality of treatment is not instituted.

- **4.E - in patients with bleeding anorectal varices, which is the appropriate pharmacological regimen (including antibiotics)?**

*In patients with anorectal varices, we suggest the use of non-selective beta-adrenergic blockers for prevention/prophylaxis of first and/or recurrent variceal bleeding (weak recommendation based on very low quality evidence, 2D). In case of acute bleeding, we suggest to temporarily suspended beta blockers (weak recommendation based on low-quality evidence, 2C).*

*In patients with bleeding anorectal varices, we suggest to consider the use of vasoactive drugs, such as terlipressin or octreotide, to reduce splanchnic blood flow and portal pressure (weak recommendation based on very low-quality evidence, 2D).*

*In patients with bleeding anorectal varices, we recommend a short course of prophylactic antibiotic (strong recommendation based on moderate quality evidence, 1B).*

The aim of medical therapy is to reduce splanchnic blood flow and portal pressure. Drugs currently in use are vasopressin, somatostatin, and terlipressin. Concomitant use of vasoactive drugs lowers portal pressure and potentially offers the endoscopist a clearer field to work in and it is the only non-invasive treatment for non-esophagogastric variceal sites of bleeding related to portal hypertension [183]. There are no trials available regarding the use of these vasoactive drugs for the treatment of bleeding ARV, but these drugs are clearly beneficial when used for acute bleeding from gastro-esophageal varices and therefore can be considered for use in bleeding ARV [141]. Vasopressin has a short half-life and should be given as a continuous *iv* infusion; furthermore, vasopressin has relevant side effects related to the important systemic vasoconstriction that could lead to mesenteric or myocardial ischemia. Therefore, vasopressin is not often used also because Terlipressin, a synthetic vasopressin analog with a longer half-life and less adverse effects, is effective in bleeding control and has a positive impact on survival. Both somatostatin and octreotide (a synthetic analog of somatostatin with longer half-life) have a good safety profile, and somatostatin is as effective as vasopressin in control of variceal bleeding. A literature review by Biecker [147] analyzed all the studies regarding the use of these drugs for the medical treatment of acute bleeding from esophageal varices and concluded that the available data is most convincing for terlipressin; however, the direct comparison of terlipressin and octreotide revealed no superiority of terlipressin. All the studies mentioned so far regard bleeding from esophageal varices, we can try to extend these indications to the varices of other districts of the body, but the grade of recommendations is necessarily weak and further studies are needed to define the best medical approach to bleeding ARV.

No trials are available regarding the role of non-selective beta-blockers and/or nitrates for the treatment of bleeding from varices other than esophageal or gastric, but non-selective beta-blockers cause vasoconstriction of the splanchnic circulation by β2-receptor inhibition and decrease cardiac output by β1-receptor blockade, and this could lead to a decrease in portal venous inflow and thereby lowers portal pressure [147]. The desired reduction of 20% in the portal pressure gradient is achieved in about 50 to 75% of patients with propranolol or carvedilol, respectively [184]. Beta-blockers have other effects besides the reduction of hepatic venous pressure gradient, such as the reduction of azygos blood flow [185, 186] and could therefore be used for the prevention of ARV bleeding. However, it should be kept in mind that hypotension (systolic blood pressure below 90 mmhg or mean arterial pressure below 65 mmhg) is a contraindication to the use of beta-blockers. Therefore, despite their prophylactic role, in the acute setting of a hypotensive patient with bleeding anorectal varices beta blockers should be temporarily suspended [184].

The abovementioned AGA guideline [175] also suggests to consider anti-fibrinolytic therapy in patients with persistent bleeding consistent with impaired clot integrity. Both ε-amino-caproic acid and tranexamic acid inhibit clot dissolution reducing hyperfibrinolysis and can be used as a rescue therapy.

Regarding antimicrobial therapy, multiple meta-analysis [187–190] showed a clear benefit in terms of survival and decrease the risk of spontaneous bacterial peritonitis when a short course of prophylactic treatment is given in all patients presenting with cirrhosis and gastrointestinal bleeding including rectal bleeding.

- **4.F - in patients with bleeding anorectal varices, what is the role for angiography?**

*In patients with bleeding anorectal varices and failure of medical treatment and local procedures, we suggest a “step up” approach with radiological and then surgical procedures (weak recommendation based on low quality evidence, 2C).*

*In patients with bleeding anorectal varices, we suggest to use embolization via interventional radiological techniques for the short-term control of bleeding (weak recommendation based on low quality evidence, 2C).*

*In patients with bleeding anorectal varices and severe portal hypertension, we suggest to use percutaneous TIPS, if not contraindicated, to decompress the portal venous system and to reduce the risk for rebleeding (weak recommendation based on low quality evidence, 2C).*

*No recommendation can be made regarding the superiority of one embolization technique over the others in case of bleeding ARV, based on the available literature.*

Embolization is performed via an interventional radiological approach and could be performed alone or in association with a TIPS procedure. The aim of embolization is to interrupt the communication between portal and systemic circulation, thus stopping the acute bleeding; however, this maneuver alone does not decompress the portal venous system and this situation lead to high rebleeding rates [179, 191–194]. The association with TIPS reduces the rates of rebleeding, decompressing the portal venous system. TIPS has the advantages of being effective and minimally invasive, it can be performed in one session and does not preclude subsequent liver transplantation; therefore it may be used during the acute situation both as a bridge to liver transplantation and as the definitive therapy in patients unfit for surgery [195]. The downsides of TIPS are the potential for increased encephalopathy (20–30%), recurrent bleeding (10%), procedure-related morbidity and a 30-day mortality of around 3–15% [141, 196]. A novel and interesting percutaneous approach is the BRTO (balloon-occluded retrograde transvenous obliteration). Developed by Kanagawa et al. In 1996 [197] for the management of gastric varices, the BRTO procedure is an endovascular technique that causes occlusion of outflow portosystemic shunt using an occlusion balloon followed by the endovascular injection of a sclerosing agent directly into the variceal system. This procedure is less invasive than TIPS and does not affect encephalopathy, but it can worsen the portal hypertension [198]. Few case series report the application of this technique to the rectum with good results in terms of hemorrhage control [182, 198]. In 2019, a case report described the combination of BRTO and TIPS, with satisfactory results [199]. Recently, some authors described a balloon-occluded anterograde transvenous sclerotherapy via direct puncture of the superior rectal vein through the greater sciatic foramen under CT guidance [200]. All the studies aforementioned are the direct demonstration that the role of intervention radiological approach is now crucial for the management of bleeding ARV, relegating surgery to a backup role in case of radiological failure.

- **4.G - in patients with bleeding anorectal varices, what are the indications for surgical treatment and what is the appropriate timing for surgery?**

*In patients with bleeding anorectal varices and failure of medical treatment, local and radiological procedures, we suggest a “step up” approach with surgical procedures (weak recommendation based on low-quality evidence, 2C).*

*In patients with bleeding anorectal varices and failure of medical treatment, local and radiological procedures, we suggest against the use of “per anal” suture ligation (weak recommendation based on very low-quality evidence, 2D).*

*No recommendation can be made regarding the role of doppler-guided hemorrhoidal artery ligation and stapled anopexy in patients with bleeding anorectal varices and failure of medical treatment, local and radiological procedures, based on the available literature.*

The current evidence supports the use of local procedures to arrest bleeding where feasible, with radiological or surgical procedures used in the event of failure. Surgical methods include simple suture ligation, inferior mesenteric vein occlusion, and porto-caval shunt surgery, but most of these patients have poor general condition and will not tolerate an invasive approach. A small retrospective case series by Bittinger et al. [201], analyzing patients with liver cirrhosis admitted for LGIB, found an 80% mortality within 2 months from bleeding for the cohort of patients with bleeding ARV, compared to a 13% mortality for patients with other sources of LGIB. The increase in mortality rate was related to hepatic failure and was not related to the hemorrhage itself. Yoshino et al. [182] conclude that a favorable prognosis may not be expected in cirrhotic patients with bleeding rectal varices, even when initial hemostasis is achieved (cumulative survival rates were 63.6% and 32.7% at 6 and 12 months, respectively) and hypothesized that hemorrhage may occur in patients with liver cirrhosis at the final stage during progression of portal hypertension. For these reasons, inferior mesenteric vein occlusion and porto-caval shunt surgery are historical, especially with the advent of percutaneous and radiological procedures.

Direct suture ligation is a technically challenging option and often not successful. Several authors have successfully used running sutures of synthetic absorbable sutures, but currently, suture ligation is not routinely advised for ARV because of the high rate of rebleeding and the difficulty in performing the procedure during active bleeding [141]. This procedure is reserved for patients who are unfit for any other procedures and in situations where no other treatment is available. The direct suture approach has been replaced in many cases by a stapled anopexy: the rationale behind this technique is the interruption of the perirectal porto-systemic anastomosis. This technique as the advantages of being easy to perform and used most colorectal surgeons for other indications, making it an available option for the initial treatment of refractory bleeding ARV. As reported by few case-reports and a small case-series, the results are encouraging and the rebleeding rate is very low [202–205]. However, stapled anopexy in cases of severe bleeding is hardly feasible and thus cannot be recommended as first-line option. A case report described the use of Doppler-guided hemorrhoidal artery ligation for the treatment of bleeding ARV [206], defining the technique as feasible and safe; unfortunately, no recommendation can be made on the base of a single-case report. In conclusion, our suggestion is to proceed with a step-up approach, starting from local endoscopic maneuvers up to TIPS or even liver transplantation in very selected cases.

5) Complicated rectal prolapse (irreducible or strangulated)
  **5.A - in patients with a suspected complicated rectal prolapse, what is the role of clinical examination and biochemical investigations?**

*In patients with suspected complicated rectal prolapse, we suggest to request complete blood count and the dosage of serum creatinine, and inflammatory markers (e.g., C-reactive protein, procalcitonin and lactates) to assess the status of the patient (weak recommendation based on low-quality evidence, 2C)*

Rectal prolapse (RP) can be defined as a circumferential, full-thickness intussusception/protrusion of the rectum through the anus. These guidelines will address only full-thickness prolapses (true or Type III) which is a complete protrusion of the rectum (and/or the sigmoid) with its entire wall through the anus [207].

An incarcerated RP corresponds at clinical examination to an external, complete RP associated with a large rectal mass or bulge that cannot be reduced manually (large, painful, immobile rectal mass). When the blood supply to the prolapsed bowel is not sufficient then the RP may become strangulated and can quickly progress to necrosis and perforation. A complete RP is a rare condition and is usually seen in extremes of life and more commonly in females [207]. In fact, the incidence of RP is approximately 2.5 per 100000 inhabitants, with a prevalence of 1% in adults over 65 years, with a women:men ratio of 9:1 [208]. Incarceration and strangulation are rare but serious and life-threatening conditions requiring prompt surgical consultation and treatment.

Rectal prolapse is generally diagnosed on the basis of patient’s full history, symptoms, and clinical examination. In clinical practice, it is essential to make differential diagnosis with prolapsed haemorrhoids: RP involves a concentric protrusion whereas prolapsed hemorrhoids are radial bulging and prolapse of discrete anal cushions. Patients admitted to ED presenting chronic or acute rectal prolapse usually complain of lower abdominal pain, constipation, and hematochezia. When a patient is admitted with a suspected diagnosis of RP, the medical history followed by a careful physical examination are usually enough to confirm the diagnosis. The decision to perform laboratory tests to assess the severity of illness should be guided by the physical examination.

Leukocytosis is usually present in patients with bowel ischemia, except in those who are immunocompromised or taking steroids, and it can be considered a potential predictor for transmural bowel necrosis, as well as a significant predictor of mortality in patients with acute mesenteric ischemia. Inflammatory biomarkers such as C-reactive protein (CRP), procalcitonin (PCT), and lactate are the most prescribed lab tests in the emergency department to evaluate the severity of the disease in case of Acute Abdomen (AA). Lactates level is a marker of poor tissue perfusion, a key element in the management of severe sepsis, septic shock, and bowel ischemia [209]. Another important test is PCT: it is known that its values are significantly correlated with intestinal necrotic damage, degree and extension of tissue damage, and mortality [210, 211]. Furthermore, there are different studies highlighting a potential role for interleukin-6 (IL-6) in the diagnostic workup of AA: it seems that its use, in combination with serum lactate, could be useful in simultaneously establishing both the severity of sepsis and the prognosis of AA [212]. In the case of a female patient of childbearing age presenting with rectal prolapse, it could be advisable to consider the dosage of β-HCG to exclude pregnancy.

- **5.B - in patients with a suspected complicated rectal prolapse, which are the appropriate imaging investigations?**

*In hemodynamically stable patients with irreducible or strangulated rectal prolapse, we suggest to perform an urgent contrast enhanced abdomino-pelvic CT-scan, whenever available and without delaying appropriate treatment, to detect possible associated complications and to assess the presence of a colorectal cancer (weak recommendation based on low-quality evidence, 2C).*

*In hemodynamically unstable patients with irreducible or strangulated rectal prolapse, we suggest against delaying appropriate and timely management to perform imaging investigations (weak recommendation based on low quality evidence, 2C).*

After assessing patient’s hemodynamic status, a contrast enhanced abdomino-pelvic CT scan should be performed to investigate potential conditions associated with complicated RP [213–216], such as acute bowel obstruction, signs of perforation and peritonitis, prolapse of other pelvic organs (uterus, vagina and/or bladder, sigmoid colon, small bowel) [217], and to rule out the presence of colorectal malignancy. A sudden RP could be the first clinical manifestation of a colon cancer, as demonstrated by several case reports [218–221]; therefore, multiple authors suggest that patients with rectal prolapse should have endoscopic examination of the colon and rectum. Akyuz et al. hypothesized that the age group in which RP is most commonly seen, the change in bowel habits, the chronic constipation, and mucosal irritation related to this disease, could be the factors that increase the incidence of rectum cancer in patients with RP and concluded that endoscopic screening should not be overlooked in this specific population [222]. An interesting retrospective study from Rashid et al. [223] compared data from 70 consecutive patients treated for RP and 350 patients of similar age treated for other conditions at a community hospital during a period of 16 years with an average follow-up of 4.4 ± 2.7 years. They reported a rectosigmoid cancer prevalence of 5.7% among patients with RP compared to a prevalence of only 1.4% in the control group. Thus, patients with RP exhibited a relative risk for colorectal cancer increased by 4.2-fold (95% confidence interval, 1.1 to 16.0, *p* < 0.02) and the authors concluded that a routinely screening of patients with symptomatic RP by use of flexible sigmoidoscopy may be appropriate.

The findings of the CT scan can guide the choice between NOM and surgery and of the best surgical approach (perineal vs. abdominal), thus reducing the risk of leaving a possible complication undetected and untreated. On the other hand, CT scan may not be promptly available in every facility, and a timely management of strangulated rectal prolapse is of utmost importance. For these reasons, the decision whether to perform a CT scan should be made based on patient clinical condition and on the available resources and should not delay the appropriate management of complicated rectal prolapse.

- **5.C - in patients with complicated rectal prolapse, what which is the role of non-operative management?**

*In patients with incarcerated rectal prolapse without signs of ischemia or perforation, we suggest to attempt conservative measures and gentle manual reduction under mild sedation or anesthesia (weak recommendation based on moderate quality evidence, 2B).*

*In hemodynamically unstable patients with complicated rectal prolapse, we suggest against delaying surgical management to attempt a conservative management (weak recommendation based on low quality evidence, 2C).*

Non-operative management (NOM) in incarcerated RP aims the following:

- ­ Reduce the edema;
- ­ Allow manual reduction;
- ­ Plan an elective definitive surgery in optimal patient’s conditions.

For irreducible RP without signs of ischemia, gentle manipulation and reduction with patient in Trendelenburg position and under intravenous sedation and analgesia is an attempt of deferring surgery to an elective setting. Every effort should be made to manually reduce an incarcerated RP in order to avoid complications such as strangulation, ulceration, bleeding, and perforation. NOM is not indicated in case of strangulated RP with signs of gangrene or perforation and in a hemodynamically unstable patient.

The techniques for NOM described in literature are as follows:

- ­ The submucosal adrenaline injections;
- ­ The topical application of granulated sugar [224]. Topical application of sugar is the most used technique because it is an easy and accessible technique to reduce edema and facilitate manual reduction of RP; the rationale behind this technique is the creation of ­ an hyperosmolar environment that attracts water molecules, thus reducing the edema of the prolapsed bowel. Unfortunately, the overall efficacy of this technique is low.
- ­ The topical application of hypertonic solutions of sugar consisting of 50% dextrose or 70% mannitol directly to the rectal mucosa by gauzes; it is based on the same mechanism of the application of sugar or salt.
- ­ The submucosal infiltration of hyaluronidase [225], which is an endoglucosidase that acts by depolymerizing hyaluronic acid causing a decomposition of the extracellular matrix. This mechanism allows the fluid collected in the third space to drain through the microscopic spaces created in the extracellular compartment thereby decreasing edema.
- ­ The elastic compression wrap [226]; this technique consists in an elastic compression band that uses increased continuous pressure to force the edema fluid out of the prolapse. This type of elastic band is often stored in operating rooms for use in orthopedic procedures and is easily accessible.

All the NOM technique should be performed with the patient in Trendelenburg position and after administration of analgesia or under mild sedation and anesthesia.

The failure rate of NOM reported in the literature for incarcerated RP is high [227], and therefore, NOM should not delay surgical treatment. Surgery should be performed when NOM fails and manual reduction is not successful, to avoid ischemia and perforation.

- **5.D - in patients with complicated rectal prolapse, what are the indications for surgical treatment and what is the appropriate timing for surgery?**

*In patients with complicated rectal prolapse and signs of shock or gangrene/perforation of prolapsed bowel, we recommend immediate surgical treatment (strong recommendation based on high quality evidence, 1A).*

*In patients with complicated rectal prolapse and bleeding, acute bowel obstruction or failure of non-operative management, we suggest urgent surgical treatment (weak recommendation based on low quality evidence, 2C).*

Immediate surgical intervention is reserved for those patients who present with incarcerated RP complicated by gangrene, perforation, or signs of shock. An urgent surgical intervention is indicated for patients with incarcerated RP associated with ulceration, bleeding, or acute bowel obstruction and failure of NOM.

There is no high-quality evidence in literature regarding the correct timing for surgical intervention after NOM failure. Again, multiple case reports highlight the relevant rate of NOM failure, delaying the surgical treatment from 4 h to 7 days from onset of symptoms [227–229]. The evidence though is not sufficient to make specific recommendations on surgical timing, except for cases with overt gangrene/perforation/shock where surgery should not be delayed.

- **5.E - in patients with complicated rectal prolapse, what is the appropriate surgical approach?**

*In patients with complicated rectal prolapse and no signs of peritonitis or hemodynamic instability, we suggest to base the decision between abdominal and perineal procedures on the specific patient’s characteristics and on surgeon’s skills and expertise (weak recommendation based on moderate quality evidence, 2B).*

*In hemodynamically stable patients with complicated rectal prolapse, in case of abdominal approach, we suggest to base the decision between open or laparoscopic surgery on patient’s characteristics and on surgeon’s skills and expertise (weak recommendation based on very low-quality evidence, 2D).*

*In patients with complicated rectal prolapse and signs of peritonitis, we suggest an abdominal approach (weak recommendation based on low-quality evidence, 2C).*

*In patients with complicated rectal prolapse and hemodynamic instability, we recommend an abdominal open approach (strong recommendation based on low-quality evidence, 1C).*

*In patients with complicated rectal prolapse undergoing resectional surgery, we suggest to base the decision between primary anastomosis, with or without diverting ostomy, and terminal colostomy on the patient’s clinical condition and on the individual risk of anastomotic leakage (weak recommendation based on low-quality evidence, 2C).*

The definitive correction of RP is surgical and multiple operative procedures are described in literature, each with their own advantages and disadvantages. In the elective setting, rectal prolapse surgical repair can be performed either through the anus (perineal approach) or through the abdomen; severity of symptoms, patient’s fitness and preferences are important factors to take into account while choosing an approach for an individual patient. Up to now, there is no proven superior technique and the various techniques can be classified based on the approach in perineal or abdominal techniques. The perineal techniques are as follows:

- Anal encirclement (Thiersch procedure)
- Mucosal sleeve resection (Delorme’s procedure)
- Perineal proctosigmoidectomy (Altemeier’s procedure)

The abdominal procedures can be performed via either an open or laparoscopic/robotic approach and are follows:

- Suture rectopexy;
- Mesh rectopexy:
  - Rectopexy with posterior fixation and anterior mesh sling (Ripstein’s procedure)
  - Rectopexy with posterior fixation and posterior mesh sling (Wells’ procedure)
  - Rectopexy with ventral fixation and double anterolateral mesh sling and modified ventral rectopexy (Orr-Loygue’s procedure)
- Resection rectopexy with or without mesh.

The debate about the optimal approach continues. To date, there is no agreement in literature whether the abdominal approach is better than the perineal one, or vice-versa. In clinical practice, in elective or emergency setting, the choice of surgical technique depends on the following:

- The patient's age and surgical risk;
- Coexisting functional symptoms (including incontinence or constipation);
- Surgeon’s familiarity with a particular surgical approach.

To our knowledge, there is no high-quality evidence regarding the best surgical approach in an emergency setting; the available data derives only from studies performed in the elective setting.

The PROSPER study [230], carried out by Senapati et al., was aimed to put an end to the debate by comparing the different procedures in the elective setting. Two hundred ninety-three patients were recruited and randomized between abdominal and perineal surgical approaches. Recurrence rates were high, but not significantly different in any comparison. The authors concluded that no significant differences were seen in any of the randomized comparisons, although substantial improvements from baseline in quality of life were noted following all procedures.

The PROSPER study was conceived after an online survey. After its publication, a further online survey (“Life after PROSPER” study) was conducted to assess if in clinical practice something was changed in the management of patients affected by RP. Interestingly, the numbers of surgeons favoring a perineal approach decreased (18.3% vs 36.5%) although the use of a perineal procedure in elderly or unfit patients was unchanged (38.5% vs 37.9%). The survey showed that the surgical management of external RP had changed and more surgeons favored a laparoscopic abdominal approach [231].

A 2015 Cochrane systematic review [232] analyzed 15 RCTs for a total of 1007 patients, to assess the optimum surgical treatment for full-thickness RP: this study showed a lack of high-quality evidences together with the small sample size of the included trials and their methodological weaknesses. The authors concluded declaring the impossibility to identify clinically important differences between the alternative surgical operations and highlighting the limit of their review for guiding practice.

In 2017, Emile et al. carried out a systematic review [233] aimed to assess the outcomes of the perineal resectional procedures, including Altemeier procedure (AP), Delorme procedure (DP), and perineal stapled prolapse resection (PSR), for the treatment of complete RP. They found that perineal resectional procedures were followed by a relatively high incidence of recurrence, yet an acceptably low complication rate, and the authors conclude that the superiority of any procedure cannot be reached due to the significant heterogeneity of the studies.

Another recently proposed option is a combined approach (laparoscopic-assisted perineal sigmoid resection with colo-anal anastomosis); Al-Ameen et al. Described this technique in a case report and highlighted the role of laparoscopy in Altemeier’s procedure for strangulated prolapse: laparoscopy allows an appropriate assessment of sigmoid length, a proper colonic mobilization, and assures that all redundant bowel is excised. They concluded that this approach could therefore reduce recurrence rate and need of further surgical interventions [234].

The only data concerning the surgical management of complicated, complete RP in emergency setting can be extrapolated from case series [235–241]. Every approach has advantages and disadvantages that should be taken into account:

- In emergency setting, perineal procedures can be performed under spinal anesthesia and are associated with lower operative morbidity and mortality (included a lower rate of damage to nerve plexus and of related sexual dysfunctions) but with higher recurrence rates than abdominal procedures; furthermore, perineal procedures require a specific set of skills and expertise. For these reasons, the perineal approach could be a feasible option for elderly or medically unfit patients when appropriate skills and expertise are available.
- Abdominal approaches have the benefit of entering the abdominal cavity, thus are the best choice in case of patients with associated peritonitis [238, 242]. Hartmann’s procedure can be an option in case of patients with rectal incontinence and in patients with signs of sepsis; moreover, Hartmann’s procedure can be safely performed by emergency general surgeons.

It is important to remember that when a colo-rectal or colo-anal anastomosis is performed, the creation of a diverting loop colostomy or ileostomy can be an option in case of increased risk of anastomotic leakage, especially in the emergency setting. In some cases, an end colostomy could be an alternative, and potentially safer, option.

- **5.F - in patients with complicated anorectal prolapse, which is the appropriate pharmacological regimen (antibiotics, pain-control, others)?**

*In patients with strangulated rectal prolapse, we suggest to administer empiric antimicrobial therapy because of the risk of intestinal bacterial translocation; the appropriate regimen should be based on the clinical condition of the patients, the individual risk for MDRO, and the local resistance epidemiology (weak recommendation based on low-quality evidence, 2C).*

According to WSES guidelines for management of intra-abdominal infections (IAI), judicious use of antimicrobials is an integral part of good clinical practice. This approach minimizes the risks associated with the selection of resistant pathogens. In critically ill patients, antimicrobial therapy should be started as soon as possible. In these patients to ensure timely and effective administration of antibiotics, always consider the pathophysiological status of the patient as well as the pharmacokinetic properties of the employed antibiotics. In patients with uncomplicated IAI, where the source of infection is treated definitively, post-operative antibiotic therapy is not necessary. In patients with complicated IAI undergoing an adequate source-control procedure, a short course of antibiotic therapy (3–5 days) is always recommended. Patients who have ongoing signs of peritonitis or systemic illness (ongoing infection) beyond 5 to 7 days of antibiotic treatment, warrant a diagnostic investigation. The choice of empiric antibiotic regimens in patients with IAI should be based on the clinical condition of the patients, the individual risk for infection by resistant pathogens, and the local resistance epidemiology. The regimen will be adapted to intra-operative cultures that allow expansion of the antimicrobial regimen if the initial choice is too narrow and to perform a de-escalation if the empirical regimen is too broad. When a microorganism is identified in clinical cultures, antimicrobial susceptibility testing should always be performed and reported to guide antibiotic therapy [49].

In the specific setting of complicated RP, the presence of strangulation or intestinal obstruction increases the risk of bacterial translocation and therefore an empiric antimicrobial therapy is suggested. After proper conservative management of incarcerated RP, there is no indication for antibiotic therapy, in absence of signs of systemic infection. In case of NOM failure and surgical treatment of RP, the antimicrobial therapy should be re-evaluated after surgery to decide, based on the intra-operative findings, whether it should be continued, discontinued, implemented, or de-escalated. Patients can also present with RP and peritonitis or sepsis/septic shock: in these cases, a proper and timely antimicrobial therapy is of utmost importance. For a throughout discussion of antimicrobial therapy in patients with IAI, we refer to WSES guidelines for management of intra-abdominal infections [48, 49].

6) Retained anorectal foreign bodies
  **6.A - in patients with suspected retained anorectal foreign body, what is the role of clinical examination and biochemical investigations?**

*In patients with suspected retained anorectal foreign body, we suggest to collect a focused medical history and to perform a complete physical examination (weak recommendation based on low-quality evidence, 2C).*

*In patients with suspected retained anorectal foreign body, we suggest to perform digital rectal examination after the acquisition of an abdomen X-ray, whenever possible, to prevent accidental injury to the surgeon from sharp objects (weak recommendation based on low-quality evidence, 2C).*

*In patients with suspected retained anorectal foreign body and no signs of bowel perforation, we suggest against routinely requesting of laboratory tests (weak recommendation based on very low-quality evidence, 2D).*

*In patients with suspected retained anorectal foreign body and no signs of bowel perforation, we suggest to request the routine preoperative blood tests only in case manual extraction fails/is not feasible (weak recommendation based on very low-quality evidence, 2D).*

*In patients with suspected retained anorectal foreign body and coexisting suspected bowel perforation, we suggest to request complete blood count, the dosage of serum creatinine, and inflammatory markers (e.g., C-reactive protein, procalcitonin, and lactates) to assess the status of the patient prior to surgery (weak recommendation based on low-quality evidence, 2C).*

Anorectal foreign bodies (AFB) are seen regularly in most large hospitals but precise epidemiological data are not available, because most of the time they are underreported. Anal erotic stimulation is by far the most common cause of retained AFB, followed by assault, accidental or iatrogenic events, ingestion of animal bones and foreign bodies, psychiatric diseases, drug trafficking, and self-treatment of fecal impaction in elderly people or prostate massage [243–245]. The male prevalence of retained AFB is striking, with a male to female ratio up to 37:1 [246–248]. The majority of patients are in their thirties [249–251], and AFB are very uncommon in children and are usually the result of assault or child abuse, which must be carefully and fully investigated [244, 251]. There is no limitation to the variety of objects found retained into the rectum [245, 252–254]: truncheon/baton, light bulbs, bottles, body spray cans, sex toys, toothbrushes, drugs, cell phones, fruits, vegetables, etc.; the wide variety of objects leads to different clinical presentations related to different degrees of damage to local tissues of the rectum and distal colon.

Typically, the hospital admission is delayed, due to the embarrassment of these patients and their effort to remove the object at home [251]; furthermore, in up to 20% of cases, patients will not initially refer to the retained AFB as the chief complaint [248]. Common complaints include rectal or abdominal pain, constipation or obstipation, bright red blood per rectum, or incontinence [255]. Given these issues, a high index of suspicion is required to accurately diagnose this condition, as well as the utmost degree of professionalism. Initial evaluation should be based on the patient’s history and physical examination; physical examination should firstly assess for the presence of peritonitis and clinical signs such as tachycardia, fever, and hypotension that should not be underestimated. Digital rectal examination is a crucial part of physical examination and provides useful information on the position of the object and the extent of local injury; however, digital rectal examination should be performed carefully and possibly after the acquisition of an abdomen X-ray, to prevent accidental injury to the surgeon from sharp objects [256, 257]. All findings collected during physical examination should be accurately reported (external aspect of the perianal area, presence of abrasions or bruising, status of the anal sphincter) because are necessary to investigate an alleged sexual assault. When these first steps do not suggest the presence of a perforation, routine laboratory exams are unnecessary, except in those cases requiring extraction under spinal or general anesthesia.

As covered in the complicated RP section, in case of suspected bowel perforation, the request of CBC and the dosage of serum creatinine, and inflammatory markers (e.g., C-reactive protein, procalcitonin and lactates) is useful to assess the general status of the patient [258]. Furthermore, serum toxicology is mandatory in cases of use or concealment of illicit drugs.

- **6.B - In patients with a suspected retained anorectal foreign body, which are the appropriate imaging investigations?**

*In patients with suspected retained anorectal foreign body, we recommend lateral and anteroposterior plain X-ray film of the chest, abdomen, and pelvis to identify the foreign body position and determine its shape, size, and location and the possible presence of pneumoperitoneum (strong recommendation based on moderate quality evidence, 1B).*

*In hemodynamically stable patients with suspected retained anorectal foreign body and a suspected perforation, we recommend a contrast-enhanced CT scan of the abdomen (strong recommendation based on moderate quality evidence, 1B).*

*In patients with retained anorectal foreign body and hemodynamic instability, we suggest against delaying surgical treatment to perform imaging investigations (weak recommendation based on low-quality evidence, 2C).*

Radiography is the major modality used in initial and follow-up imaging of foreign bodies. When foreign bodies are evaluated on radiographs, it is important to remember that they have different radiopacity and radiographic visibility, related to the X-ray attenuation characteristics of the object, its surrounding structures, and the overlying and underlying structures [257]. A biplanar plain X-ray of the chest, abdomen, and pelvis can detect the majority of retained AFB and gives useful information regarding the number, size, shape, location, and orientation of the foreign bodies [248, 259, 260] and possible presence of pneumoperitoneum. The plain X-ray could help predict the cases in whom transanal extraction is likely to be unsuccessful: in fact, the distinction between high- or low-lying retained objects, the latter being usually amenable of manual extraction in the ED, can be usually achieved with abdominal X-rays [249, 261]. However, non-visualization of any object on X-rays does not rule out the presence of retained AFB, because some foreign bodies have very low radiopacity and radiographic visibility. A contrast-enhanced CT scan is usually the next step and is helpful in locating non-radiopaque objects, in case of suspected bowel-free perforation or abscess or in the diagnosis of bowel obstruction; in fact, CT scan allows to evaluate not only the foreign body but also its related complications [257]. In case a CT scan is not available, erect or lateral decubitus chest radiographs can be performed to evaluate the presence of pneumoperitoneum. Erect and left lateral decubitus X-rays have similar diagnostic accuracy, the latter being better tolerated by patients presenting with peritonitis. Water-soluble contrast enema is another valuable option in case of unavailability of CT scan and can be used to evaluate complications such as rectal perforation or fistula [257]. “Point-of-care” ultrasound could also detect free intra-peritoneal, when performed by a trained operator, but it is highly operator dependent and some ultrasound machines have low-quality images; furthermore, sonography has low reliability in obese patients and in case of subcutaneous emphysema [262]. For these reasons, its role in the diagnostic workup of AFB still needs to be defined. Some authors also advocate the execution of a repeated plain film of the abdomen after the extraction process, to ensure that no perforation took place [243, 263]. Given the low sensitivity of the plain X-ray for the detection of pneumoperitoneum, this routine use of repeated radiograph can even be deleterious in case of false negative findings.

- **6.C - in patients with retained anorectal foreign body, what is the most appropriate interventional approach (manual extraction vs endoscopy vs surgery)?**

*In patients with low-lying retained anorectal foreign body without sign of perforation, we suggest an attempt of bedside extraction as the first-line therapy (weak recommendation based on low-quality evidence, 2C).*

*No recommendation can be made regarding the superiority of one trans-anal extraction technique over the others in case of retained anorectal foreign body, based on the available literature.*

*In patients with retained anorectal foreign body and failure of bedside extraction, we suggest pudendal nerve block, spinal anesthesia, intravenous conscious sedation, or general anesthesia to improve chances of transanal retrieval (weak recommendation based on low-quality evidence, 2C).*

*In patients with retained high-lying anorectal foreign body (above rectosigmoid junction), we suggest an attempt of endoscopic extraction as the first-line therapy (weak recommendation based on low-quality evidence, 2C).*

*In patients with retained anorectal foreign body and suspect of drug concealment, we suggest against any maneuver that can disrupt the drug package, including endoscopic retrieval (weak recommendation based on low-quality evidence, 2C).*

*In patients with retained anorectal foreign body, we suggest to perform a proctoscopy or flexible sigmoidoscopy after foreign body removal, to evaluate bowel wall status (weak recommendation based on low-quality evidence, 2C).*

*In patients with retained anorectal foreign body and signs and hemodynamic instability or perforation, we recommend against transanal extraction (strong recommendation based on low-quality evidence, 1C).*

The treatment of a retained AFB depends firstly on the patient’s clinical situation: if peritonitis or signs of pneumoperitoneum are present any attempt of bedside extraction is contraindicated and the patient should undergo urgent surgical exploration, after an initial resuscitation with intravenous fluids and administration of intravenous antibiotics; if the patient is unstable, emergent laparotomy should be performed. Other factors that affect the treatment are the location of the AFB (objects in the sigmoid colon are more likely to require operative extraction compared with those in the rectum), the type of object (shape and size), and the number of objects [249]. The removal of small, non-sharp, and low-lying AFBs can be performed safely in the ED, while bigger, sharp, or high-lying AFBs requires a prompt referral to the specialist surgical team to warrant a safe extraction in the OR. The reviews currently available in literature report that transanal extraction is successful in 60 to 75% of cases [248, 250, 255]. The use of anesthesia (including pudendal nerve block, spinal anesthesia, or intravenous conscious sedation) helps to relax the patient, reduces anal sphincter spasm, improves visualization, and increases the chances of successful transanal retrieval. Multiple instruments designed for other purposes can be used for transanal retrieval of AFB, and they can act either by grasping the foreign body or by scooping it out from the rectum, but based on the available literature, no recommendation can be made regarding the superiority of one instrument/technique over the others [245, 248, 255].

The use of post-extraction procto-sigmoidoscopy is debated; the rationale behind this further examination is to check the condition of the bowel wall after the retrieval maneuvers and to identify supernumerary AFB or fragments of the removed AFB. While some studies advocate that clinical observation is enough in all those cases where a perforation was not suspected before extraction [249], other authors reported a significant rate of post-extraction perforations [248] and recommend a routinely use of endoscopy after AFB extraction [245, 251, 255]. Given the paucity of good quality data and the relative safety of the endoscopic examination, we cannot make a strong recommendation, but we suggest the use of post-extraction endoscopy.

Rigid or flexible sigmoidoscopy should be performed to visualize and attempt extraction of all the high-lying AFBs that are out of reach and sight with a manual transanal approach. Multiple techniques are described in literature (polypectomy snare, endoscopic grasper or net, biopsy forceps, guidewire, and balloon dilator [264]), and the addition of fluoroscopy can be helpful to assist in the removal of the object. Thus, endoscopy could be considered “the next-step” after unsuccessful transanal extraction of low-lying object and for all the high-lying AFB that are out of reach for manual transanal extraction. A specific subset of patients who conceal drugs as retained AFB, the “body packers” [265], require modified application of these indications and should be managed with great caution, because in the event of perforation or damage to the drug package the patient can experience the life-threatening complications of drug overdose. For this reason, endoscopic retrieval is contraindicated by different authors and specialist societies [266–268]. It is thus important to keep in mind the possibility of encountering a drug pack inside the rectum and should this be the case, every maneuver that can cause the disruption of the drug package should be avoided. Furthermore, when drug packages are detected in the anorectal region, a complete survey of the GI tract is required as many of these are swallowed rather than inserted retrograde.

- **6.D - in patients with retained anorectal foreign body, what are the indications for surgical treatment and what is the appropriate timing for surgery?**

*In patients with retained anorectal foreign body and no signs of perforation, we suggest a surgical approach in case of failure of transanal extraction (weak recommendation based on low-quality evidence, 2C).*

*In patients with retained anorectal foreign body and no signs of perforation, we suggest a “step-up” surgical approach, starting with downward milking and proceeding to colotomy only when milking/transanal extraction fails (weak recommendation based on low-quality evidence, 2C).*

*In patients with retained anorectal foreign body and no signs of perforation, we suggest a laparoscopic approach if skills and instrumentation are available (weak recommendation based on low quality evidence, 2C).*

*In patients with retained anorectal foreign body and bowel perforation with limited peritoneal contamination, we suggest primary suture only in case of small and recent perforation and if the colonic tissues appear healthy and well vascularized, and an approximation of perforation edges could be performed without tension (weak recommendation based on low-quality evidence, 2C).*

*In patients with retained anorectal foreign body and bowel perforation, clinically stable and without risk factors for anastomotic leakage, when primary suture is not feasible, we suggest resection with primary anastomosis with or without a diverting stoma (weak recommendation based on low-quality evidence, 2C).*

*In critically ill patients with retained anorectal foreign body and bowel perforation, or in selected patients with extensive peritoneal contamination and risk factors for anastomotic leakage, we suggest to perform a Hartmann’s procedure (weak recommendation based on low-quality evidence, 2C).*

*In patients with retained anorectal foreign body and hemodynamic instability, we recommend an emergent laparotomy and a damage control surgery approach (strong recommendation based on moderate quality evidence, 1B).*

Lake et al. [249] analyzed the predicting factors for operative intervention in case of retained AFB and found that the only factor that is statistically related to transanal extraction failure is migration of AFB into the sigmoid colon, that increases the risk of undergoing to operative intervention by 2.25 fold. After failure of transanal retrieval, the patient should undergo general anesthesia and a repeated rectal examination under anesthesia should be attempted, as the complete relaxation subsequent to muscular paralysis may allow transanal extraction. If this attempt fails, then surgery is the following step. However, some authors state that surgery should be considered as the first-line treatment in case of high-lying, hard, or sharp-edged objects [269]. Surgery in stable patients is guided by a step-up approach. Laparoscopy should be performed to begin with, as it allows to evaluate the contamination of the peritoneal cavity and, in the absence of a perforation, a laparoscopic-assisted technique can be performed, gently milking downward the AFB to allow a transanal extraction [253, 270]. Milking can be performed manipulating the object distally using transmural pressure and, wherever feasible, is preferred to colotomy. In fact, colotomy should be reserved to those cases where all the abovementioned procedures fail. All these maneuvers can also be accomplished via a midline minilaparotomy, in case the laparoscopic skills and instrumentation are not available. The rate of perforation in case of retained AFB is around 15% [271, 272]. In case of perforation, the surgical options vary depending on patient’s status, degree of peritoneal contamination, duration of the perforation, and status of the bowel walls. A small, fresh perforation with limited peritoneal contamination can be treated as a iatrogenic perforation and can undergo primary repair after retrieval of the foreign object, if the colonic tissues appear healthy and well vascularized, and an approximation of perforation edges could be done without tension [273]. Hartmann’s procedure has been considered the procedure of choice in patients with generalized peritonitis; it remains a safe technique for emergency colectomy in case of diffuse peritonitis, and it is especially useful in critically ill patients and in patients with multiple comorbidities. However, restoration of bowel continuity after a Hartmann’s procedure is associated with significant morbidity and resource utilization and many of these patients do not undergo reversal surgery, remaining with a permanent stoma [274, 275]. Given the absence of high-quality evidence in the specific population of AFB, we refer to the trauma literature [276–279] and to the WSES guidelines for the management of acute colonic diverticulitis [280] and for the management of iatrogenic colonoscopy perforation [281]: we suggest that a resection with primary anastomosis, with or without a diverting stoma, may be performed in otherwise healthy patients, with good tissue quality and without risk factors for anastomotic leakage, in case a primary suture is not feasible. On the other hand, Hartmann’s procedure is suggested for the management diffuse peritonitis in critically ill patients and in selected patients with multiple comorbidities and risk factors for anastomotic leakage (i.e., requirement of vasoactive drugs, hemodynamic instability, corticosteroid therapy). In case of unstable patients, an emergent laparotomy is mandatory and should be guided by the damage control surgery principles.

- **6.E - in patients with retained anorectal foreign body is there a role for antibiotic therapy?**

*In patients with retained anorectal foreign body, we suggest against the routine use of antimicrobial therapy (weak recommendation based on low-quality evidence, 2C).*

*In patients with retained anorectal foreign body and signs of hemodynamic instability or perforation, we recommend broad spectrum antibiotic therapy according to the WSES guidelines on intra-abdominal infections (strong recommendation based on moderate quality evidence, 1B).*

Based on the available literature, no data regarding the role of antibiotics in patients with retained AFB are available. In the light of the steady increase of antibiotic resistances globally, we suggest against the use of antimicrobial therapy for patients without sign of infection or perforation. For patients with AFB and perforation, peritonitis, or other septic complications, we refer to WSES guidelines for management of intra-abdominal infections for further information [49, 282].

7) Acute anal fissure
  **7.A - in patients with suspected acute anal fissure, what is the role of clinical examination and biochemical investigations?**

*No recommendation can be made regarding the role of biochemical investigations in patients with typical acute anal fissure, based on the available literature.*

*In patients with atypical acute anal fissure, we suggest to collect a focused medical history and perform a complete physical examination and laboratory tests based on the suspected associated illness, to rule out other causes (weak recommendation based on low-quality evidence, 2C).*

Anal fissure (AF) is a longitudinal tear within the anal canal that can extend from the dentate line to the anal verge. The diagnosis of AF commonly occurs in the third decade of life without any difference among sex [283]. Approximately 90% of anal fissures are located posteriorly in the midline, while anterior fissures occur in 10% of women versus 1% of men [284]. AF are rarely located laterally in the anal canal or multiple in number. In these atypical cases, associated diseases such as inflammatory bowel disease (IBD), sexually transmitted diseases (HIV, syphilis, or herpes), anorectal cancer, or tuberculosis should be ruled out [285, 286]. The exact etiology of AF is still unknown. Mechanical rauma caused by the passage of hard stools is not enough to explain their onset and less than 25% of patients with AF complains of constipation. Moreover, manometric studies have supported the ischemic ulcer theory, with a strong correlation between internal anal sphincter (IAS) hypertonia and decreased anodermal vascular blood flow [287].

AF can be divided, temporally and morphologically, into acute and chronic: acute fissures are superficial, with well-demarcated edges and last less than 6 weeks; chronic fissures are characterized by a distal sentinel tag and a proximal hypertrophied anal papilla with the exposure of the IAS fibers at the base of the AF and symptoms for over 6 weeks [288].

The diagnosis of AF is mainly clinical and is based on recent and past medical history, including family history and complete physical examination. Patients with AF often complain of severe and prolonged anal pain, referred as passing a “broken glass”, during and after defecation [289]. Other associated symptoms include spasm and/or intermittent bleeding with defecation. When the tone of the IAS is too high, digital rectal examination can be painful and an examination under anesthesia is indispensable to rule out other concomitant diseases such as anal abscess or fistula [290]. No recommendation can be made regarding the role of biochemical investigations among patients presenting typical acute anal fissure or referring similar symptomatology, based on the available literature. In case of a suspicion but non-conclusive physical examination, when concomitant diseases are suspected, laboratory tests should be based on the supposed associated diseases to rule out other causes.

- **7.B - in patients with suspected acute anal fissure, which are the appropriate imaging investigations?**

*No recommendation can be made regarding the use of imaging investigations in patients with typical acute anal fissure, based on the available literature.*

*In patients with atypical acute anal fissure, we suggest to perform investigations (endoscopy, CT scan, MRI, or endoanal ultrasound) only in case of suspected concomitant inflammatory bowel disease, anal or colorectal cancer or occult perianal sepsis (weak recommendation based on low-quality evidence, 2C).*

Usually, no imaging investigations are necessary at initial presentation of acute AF. In case of a suspicion but non-conclusive physical examination, if the fissure cannot be seen, the diagnosis is unclear,  or if there is significant bright red bleeding in a patient with an increased risk for colorectal cancer or in patients above 50 years of age or if there are features suggesting a secondary anal fissure, imaging such as endo-anal ultrasound, endoscopy, CT scan, or MRI, may be required [285, 286]. The choice of the appropriate imaging technique should be driven by the suspected underling disease. Anorectal manometry could be indicated in patients who have undergone previous surgery, but it is frequently too painful to be performed with in the emergency setting.

- **7.C - in patients with an acute anal fissure, what is the role of non-operative management?**

*In patients with acute anal fissure, we recommend non-operative management as the first-line treatment (strong recommendation based on moderate-quality evidence, 1B).*

*In patients with acute anal fissure, we recommend dietary and lifestyle changes, with increased fiber and water intake (strong recommendation based on moderate quality evidences, 1B).*

*In patients with acute anal fissure, we recommend against the use of manual dilatation (strong recommendation based on moderate quality evidences, 1B).*

*No recommendation can be made regarding the use of controlled anal dilatation in patients with acute anal fissure, based on the available literature.*

Initial treatment of anal fissures is medical or non-operative. There are only few studies concerning non-operative management in the treatment of acute AF: 50% of patients will not require further therapy solving the problem, in 10–14 days, with non-operative measures such as sitz baths, fiber, and topical agents [291–295]. However, to prevent a recurrence of AF, the combined use of dietary and lifestyle changes and medical therapy are recommended.

The primary goals of AF therapy are to achieve IAS relaxation, thus reducing pain and facilitating the healing process; minimize anal trauma; increase blood flow; and treat pain. To obtain these results the cornerstones of NOM are as follows:

- *Stool softeners* (increased intake of oral fluids, high-fiber diet or fiber supplements, and bulk forming laxatives);
- *Sphincter muscle relaxers* (warm sitz baths, local application of calcium channel blockers like Diltiazem or Nifedipine, local application of Nitrates like Nitroglycerin and Botulinum injection that determines a temporal paralysis of the anal sphincter muscle for 2–3 months). As already mentioned above, the literature regarding acute AF is scarce and the available data are often mixed with those regarding chronic AF, and this is especially true regarding muscle relaxers. Several pharmacological agents have been used with the purpose of inducing the so-called chemical sphincterotomy: based on the pathophysiology of the disease, NO donors (glyceryl trinitrate) are vasodilator and may support AF healing; they promote an increase in local blood flow and reduce IAS tone. On the other hand, calcium channel blockers (CCB) block slow L-type calcium channels of vascular smooth muscle cells, thus reducing the IAS tone and promoting an increase in local blood flow. In a single double-blind, randomized, prospective trial on 110 patients with chronic AF, Perrotti et al. [296] demonstrated the effectiveness of an industrially manufactured ointment (0.3% nifedipine; 1.5% lidocaine). The authors reported a healing rate of 95% after 6 weeks compared to 16% of control group (1% hydrocortisone and 1.5% lidocaine). According to a recent systematic review and meta-analysis, including 148 trials and 29 different non-surgical treatments, CCBs (diltiazem or nifedipine) were more effective than glyceryl trinitrate and with less risk of headache and hypotension [295]. In particular, CCBs are associated with a healing rate ranging from 65 to 95% [297, 298] and with a remarkable cost-effectiveness compared to other non-operative treatments [299]. Furthermore, considering the possibility of systemic side-effects and the similar rates of healing and pain relief [300], the topical use of CCBs is suggested.
- *Pain control* (see question 7.D).

There is no standard duration of therapy but administration for at least 6 weeks is suggested, with pain relief occurring usually after 14 days [296, 301].

The hypertonicity of internal anal sphincter was historically treated with manual dilatation. This practice was abandoned [302], due to the high risk of incontinence (temporary and permanent incontinence rates can reach 30% and 10%, respectively [303, 304]) and the superiority of internal sphincterotomy [295, 305, 306]. Nevertheless, lateral internal sphincterotomy has its own wound-related complications including fistula, bleeding, abscess, or non-healing wound in up to 3% of patients [307, 308]. For these reasons, less traumatic, precise, measurable, and reproducible techniques of anal dilatation were recently designed and introduced into practice: balloon dilatation and staged dilatation showed healing rates superimposable to those obtained after internal sphincterotomy, with complication and incontinence rates near zero [309–314]. These results were recently confirmed in a long-term follow-up study [315]. Unfortunately, all the studies cited above are mainly focused on chronic anal fissures. For these reasons, controlled anal dilatation could be included in the non-operative management and could be taken into account before surgical treatment of chronic anal fissure, but the available data are not enough to make recommendation in the acute setting.

- **7.D - in patients with an acute anal fissure, what is the appropriate approach for pain control?**

*In patients with acute anal fissure, we suggest the integration of topical anesthetics and common pain killers in case of inadequate pain control (weak recommendation based on low-quality evidences, 2C).*

*No recommendation can be made regarding the use of botulinum injections in patients with acute anal fissure, based on the available literature.*

Adequate pain control is a cornerstone of acute AF management; the absence of pain helps reducing the tone of IAS and promotes correct bowel habits, preventing the excruciating pain associated with defecation. In fact, analgesics relief the constant reflex spasm of the anal sphincter and thus reducing local ischemia and enhancing fissure healing. Lidocaine is the most commonly prescribed topical anesthetic for anal fissures, while administration of pain killers like paracetamol or ibuprofen (either oral or parenteral) and/or perianal infiltration of anesthetics are indicated for patients with severe acute pain.

The mechanism of Botulinum toxin in striated muscle is well known: it acts on the presynaptic nerve terminal of the neuromuscular junction reducing the release of acetylcholine [316]. Several studies investigated the role of Botulinum toxin on AF, demonstrating the effectiveness in promoting healing of AF refractory to the first-line treatment with CCB or glyceryl trinitrate. However, there are still controversies regarding the dose, preparation, and the site of injection. Only one study by Othman et al. [317] focused on the treatment of acute AF. The authors compared the bilateral (group 1) or posterior (group 2) use of Botulinum toxin in patients with AF. In both groups healing of the AF has been reached in 5.20 and 5.40 weeks, respectively, whereas pain relief occurred earlier in group 2 (8.45 vs 7.2 days). The authors concluded that the use of a single posterior injection is more tolerable and less painful than bilateral injection [317]. Few other studies analyzed the role of Botulinum toxin for the treatment of chronic AF [318, 319]. The results of these studies regarding the dose, preparation, and the site of injection are interesting but cannot be extended to the treatment of acute anal fissures.

- **7.E - in patients with an acute anal fissure, is there a role for antibiotic therapy?**

*In patients with acute anal fissure, we suggest the use of topical antibiotics in case of potential reduced therapeutic compliance or poor genital hygiene (weak recommendation based on very low-quality evidences, 2D).*

According to Garg, the use of antibiotics should be reserved for chronic or acute-on-chronic AF in which there can be a low-grade infection [320]. In a recent prospective controlled randomized study [321], 100 patients with acute AF were randomly divided into two groups (group 1: 5% lidocaine; group 2: 5% lidocaine plus metronidazole cream). All patients applied the therapy 3 times per day for 4 weeks. Pain was assessed using a visual analog scale. At the end of 2nd and 4th week, there was a statistically significant difference between the two groups and in both cases in favor of group 2 (2 weeks 3.3 vs 2.6, *p* = 0.004;4 weeks 2.47 vs 1.36, *p* < 0.001). The final healing rate was 56% in group 1 and 86% in group 2. The authors suggested the use of metrodinazole in addition to the traditional therapies.

However, considering the methodological limitations of the study and the absence of additional evidence, the recommendations on the use of antibiotics in the treatment of acute AF is weak.

- **7.F - in patients with an acute anal fissure, what are the indications for surgical treatment and what is the appropriate timing for surgery? If indicated, what is the most appropriate surgical approach?**

*In patients with acute anal fissure, we suggest against surgical treatment (weak recommendation based on moderate quality evidences, 2B).*

*In patients with anal fissure, we suggest surgical treatment in the chronic phase, if non-responsive after 8 weeks non-operative management (strong recommendation based on moderate quality evidences, 1B).*

Patients with an acute AF should be managed with a combination of dietary and lifestyle modification and medical therapy. A surgical approach is suggested only in the chronic phase, after the failure of 6–8 weeks of NOM. In this context, lateral internal sphincterotomy is the preferred technique with a lower recurrence rate, a higher patient satisfaction [295, 305], and a healing rate of over 90% [322]. Open and closed lateral internal sphincterotomy have similar results although in a prospective randomized study, open lateral internal sphincterotomy was associated with higher post-operative pain and a delayed wound healing at 1 year [323]. Both techniques are superior with respect to fissurectomy and posterior sphincterotomy in terms of healing rate, post-operative pain, and fecal incontinence.

## Conclusions

Anorectal emergencies are common causes of ED referral and comprise a great variety of diseases, ranging from benign to life-threatening. Currently, there are no guidelines available in the literature addressing the specific setting of acute care surgery for anorectal disease. For these reasons, these guidelines present evidence-based international consensus statements on the management of anorectal emergencies from collaboration of a panel of experts and are intended to improve the knowledge and the awareness of physicians around the world on this specific topic. The structure of the guidelines, divided into seven main topics that cover the entire management process of patients with anorectal emergencies, provides an up-to-date, easy-to-use tool that can help physicians and surgeons during the decision-making process.

## Data Availability

The authors are responsible for the data described in the manuscript and assure full availability of the study material upon request to the corresponding author.

## Abbreviations

AA: Acute abdomen
AAST: American Association for the Surgery of Trauma
AF: Anal fissure
AFB: Anorectal foreign bodies
AGA: American Gastroenterology Association
ARV: Anorectal Varices
ASA score: American Society of Anesthesiologists Score
BRTO: Balloon-occluded retrograde transvenous obliteration
CBC: Complete blood count
CCB: Calcium channel blockers
CPR: C-reactive protein
CRC: Colorectal cancer
ED: Emergency department
EUS: Endoscopic ultrasound
FGSI: Fournier’s Gangrene Severity Index
GRADE: Grading of Recommendations Assessment, Development and Evaluation
IAI: Intra-abdominal infection
IAS: Internal anal sphincter
IBD: Inflammatory bowel disease
IL-6: Interleukin-6
LGIB: Lower gastro-intestinal bleeding
LRINEC: Laboratory Risk Indicator for Necrotising Fasciitis
MDRO: Multidrug-resistant organism
MRI: Magnetic resonance imaging
MRSA: Methicillin-resistant *Staphylococcus aureus*
NOM: Non-operative management
NPWT: Negative pressure wound therapy
OR: Operating room
PCT: Procalcitonin
RP: Rectal prolapse
SFGSI: Simplified Fournier’s Gangrene Severity Index
SIARI score: Site other than lower limb, immunosuppression, age < 60 years, renal impairment, inflammatory markers
TIPS: Transjugular intrahepatic portosystemic shunt
UFGSI: Uludag Fournier’s Gangrene Severity Index
WSES: World Society of Emergency Surgery
